# Spectroscopic Insights of an Emissive Complex between 4′-*N*,*N*-Diethylaminoflavonol in Octa-Acid Deep-Cavity Cavitand and Rhodamine 6G

**DOI:** 10.3390/molecules28114260

**Published:** 2023-05-23

**Authors:** Fabiano da Silveira Santos, Elamparuthi Ramasamy, Lilian Camargo da Luz, Vaidhyanathan Ramamurthy, Fabiano Severo Rodembusch

**Affiliations:** 1 Grupo de Pesquisa em Fotoquímica Orgânica Aplicada, Instituto de Química, Universidade Federal do Rio Grande do Sul, Av. Bento Gonçalves 9500, Bairro Agronomia, Porto Alegre CEP 91501-970, Brazil; fabiano@ufrgs.br (F.d.S.S.);; 2 Department of Chemistry and Biochemistry, The University of Texas at Arlington, Arlington, TX 76019, USA; 3 Department of Chemistry, University of Miami, 1301 Memorial Drive, Miami, FL 33124, USA

**Keywords:** supramolecular photochemistry, emissive complex, octa acid, flavonol, static quenching, time-resolved fluorescence

## Abstract

Excited-state chemistry relies on the communication between molecules, making it a crucial aspect of the field. One important question that arises is whether intermolecular communication and its rate can be modified when a molecule is confined. To explore the interaction in such systems, we investigated the ground and excited states of 4′-*N*,*N*-diethylaminoflavonol (DEA3HF) in an octa acid-based (OA) confined medium and in ethanolic solution, both in the presence of Rhodamine 6G (R6G). Despite the observed spectral overlap between the flavonol emission and the R6G absorption, as well as the fluorescence quenching of the flavonol in the presence of R6G, the almost constant fluorescence lifetime at different amounts of R6G discards the presence of FRET in the studied systems. Steady-state and time-resolved fluorescence indicate the formation of an emissive complex between the proton transfer dye encapsulated within water-soluble supramolecular host octa acid (DEA3HF@(OA)_2_) and R6G. A similar result was observed between DEA3HF:R6G in ethanolic solution. The respective Stern–Volmer plots corroborate with these observations, suggesting a static quenching mechanism for both systems.

## 1. Introduction

**Figure 1:**
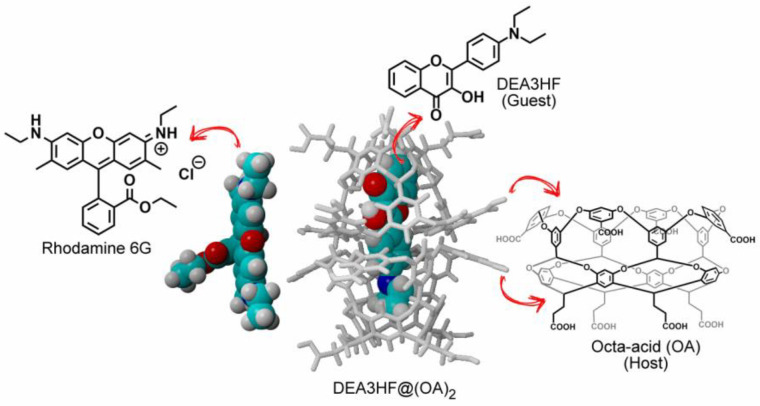
Chemical structure of DEA3HF (guest), octa acid (host), DEA3HF@(OA)_2_, and Rhodamine 6G (R6G).

Supramolecular chemistry is based on intermolecular interactions, where building blocks are linked together through non-covalent bonds [1]. Closely linked to the definition is the term host–guest chemistry, as established by Cram [2,3]. The same forces are at play where the host is a macromolecule and the guest molecule is the smaller of the two. The dynamic nature of the non-covalent interactions and selective host–guest complexation give the resulting nanomaterials intriguing properties, holding promising potential in several fields, especially in biomedical applications [4,5,6,7]. This interest has resulted in the understanding and exploration of a variety of macrocycles, such as cyclodextrins [8], calixarenes [9], cucurbiturils [10], and pillarenes [11]. In this sense, the study of the intermolecular interaction in supramolecular architectures is worth investigating, since it may elucidate fluorescence quenching mechanisms that may involve electron or energy transfer, which can have potential for applications in photonics and electronics [12,13]. In this supramolecular context, host-type octa acids (OA) [14,15] have been attracting interest due to their properties to host photoactive molecules [12,16,17,18,19,20]. It is also called a deep cavity due to its greater depth when compared to other macrocycles [21,22,23], where its structure features a hydrophobic inner pocket and an outer coating with eight carboxylic acids in the upper and lower rims of this host, making it soluble in water under basic conditions (pH~9.0) [23,24,25,26,27]. Depending on factors, such as the hydrophobicity and size of the guest [27,28], OA has the interesting ability to form either a complex from an open cavitandplex, or a closed capsuleplex. In different proportions, it can be to form 1:1, 2:1, or 2:2 host-to-guest complexes [29,30,31]. In previous work [32], we studied the influence of the confined environment on 3-hydroxyflavone (3HF) and 4′-*N*,*N*-diethylaminoflavonol (DEA3HF), which can proton–transfer in the excited state by the ESIPT (Excited State Intramolecular Proton Transfer) process. In this study, we observed that the dyes had a photophysical behavior close to that observed in an organic solvent solution; even encapsulated and solubilized in water, important properties were preserved, such as photochemical and photophysical stability and a large Stokes shift [33]. The solubility of organic dyes is often limited to organic solvents, and the use of the supramolecular approach, which involves confining a capsule with a water-soluble hydrophobic interior, using the OA capsule as the medium for this, significantly expands the study and applications of fluorescent compounds [34,35,36]. Regarding energy transfer in this confined media, the literature reports the interaction study of coumarin dye in OA complexes with R6G as an acceptor [16]. In this investigation, the R6G, with charge +1, is electrostatically associated with octa acid, but ruling out the possibility of inclusion complex formation [16,37]. In addition, it was observed that R6G does not form an inclusion complex with OA, remaining in solution, which is associated with the exterior of the OA capsule. As pointed out by Gupta et al., these results suggest that the donor alone in a cavity is not being quenched by the acceptor dominating the steady-state fluorescence. This latter could be probably related to the occurrence of ultrafast Förster resonance energy transfer (FRET) between the donor and the acceptor through the walls of octa acid, as observed in coumarin dyes@(OA)_2_:R6G [16]. Förster resonance energy transfer (FRET) is the radiation-less energy transfer from an excited donor molecule to a suitable ground-state acceptor molecule via through-space resonant dipole coupling [38,39]. FRET occurs between an excited donor molecule (D) and the ground-state acceptor molecule (A) over a range of distances, typically 10–100 Å. The FRET efficiency is strongly dependent on the D-A distance and is characterized by the Förster critical radius R_0_, a unique parameter for each D-A pair. For this type of process to occur, some conditions are required, such as the resonance of the oscillations of the electric fields of the excited state of the donor and the fundamental state of the receiver, in addition to the spectral overlap between the emission of the donor and the absorption of the receiver, and a spatial orientation between the electric dipoles of the two states directly involved [38,40]. Recent studies explored FRET in a confined environment and demonstrated how these guest–host building blocks result in the energy transfer between the donor and acceptor [16,41,42,43,44,45,46]. Based on these observations, in this work, we aimed to broaden the knowledge regarding the intermolecular interaction between a flavonol in confined media, the guest–host system (DEA3HF@(OA)_2_), and R6G (Figure 1) by steady-state and time-resolved spectroscopies. Finally, the system DEA3HF:R6G in ethanol was also investigated for comparison purposes.

## 2. Results and Discussion

**Figure 2:**
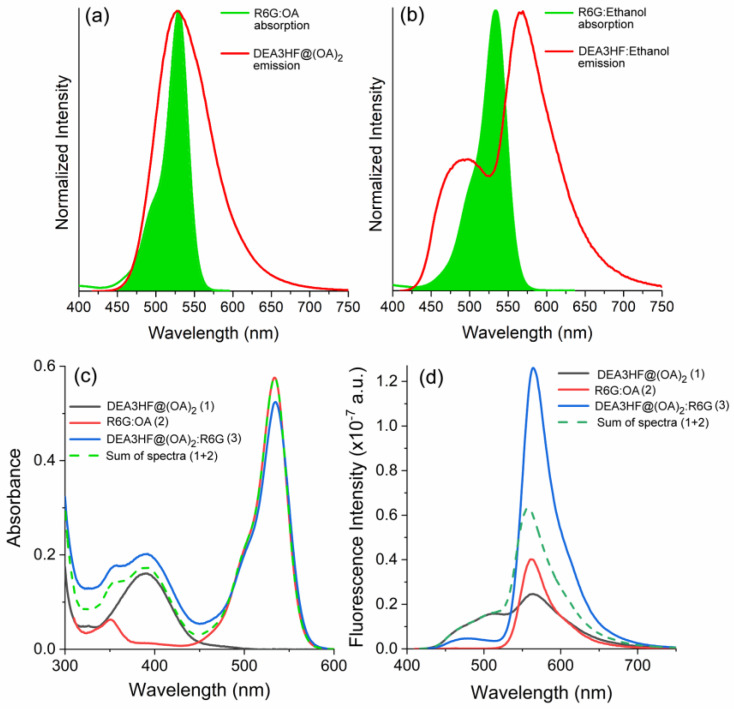
Spectral overlap for (**a**) DEA3HF@(OA)_2_:R6G in sodium borate buffer and (**b**) DEA3HF:R6G in ethanol. (**c**) UV-Vis absorption and (**d**) steady-state fluorescence emission spectra (λ_exc_ = 405 nm) in sodium tetraborate buffer of DEA3HF@(OA)_2_, R6G in the presence of OA, and DEA3HF@(OA)_2_ in the presence of R6G. The sum of DEA3HF@(OA)_2_ and R6G spectra was presented for comparison purposes (dash line).

The investigation of the interaction between the proposed flavonol (DEA3HF@(OA)_2_ and DEA3HF in ethanol) and R6G was first explored by taking their spectral overlap into account. This parameter is fundamental to discussing intermolecular interactions since it can be related to the extent of energy transfer in the excited state, and consequently to the fluorescence quenching mechanism [47]. Experimentally, the spectral overlap of the initially excited and equilibrium emissions can easily allow a solid choice between different models for spectral relaxation [48]. Although the spectral overlap does not imply energy transfer, the overlap between the absorption spectrum of an acceptor with the fluorescence emission spectrum of a donor is a sine qua non condition, for instance, to Förster resonance energy transfer (FRET), but is not sufficient for FRET [38,39,40]. In this sense, to investigate FRET in these systems, the separated photophysical characterization of both the flavonol (acting as the donor) and R6G (acting as the acceptor) was performed in the studied media. Firstly, since R6G was used as the acceptor in both systems, its absorption spectra were acquired in different environments (ethanol, water, and borate buffer in the presence and absence of OA). In general, R6G showed absorption curves between 450–575 nm with maxima at ~530 nm (Figure S1), where no significant changes were observed [49]. In addition, the UV-Vis absorption of DEA3HF@(OA)_2_ in borate buffer and the free DEA3HF in ethanol, as well as the fluorescence emission spectra of the flavonol and rhodamine in all studied media, were obtained (Figure S2). The DEA3HF in ethanol showed an absorption maximum of 412 nm and a single fluorescence emission of around 520 nm. This latter is related to intermolecular hydrogen bonding with the solvent [32]. On the other hand, in confined media, its absorption is located at 400 nm and shows a dual fluorescence emission between 450–700 nm. In this particular case, the observed dual emission is due to the normal species (N*) with a charge–transfer character (~475 nm), and another one arises from the emission of the tautomeric species (T*) (~575 nm) [32]. The R6G in both ethanol and borate buffer environments showed emission around 560 nm. Finally, after merging the emission spectra of the DEA3HF with the absorption spectra of R6G, the spectral overlap (green area) could be observed in both proposed systems, as depicted in Figure 2a,b. Quantitatively, the overlap analysis (OriginLab^®^) indicated that the integral of the spectral overlap in DEA3HF:R6G in ethanol is ~15% lower (35.07) than in DEA3HF@(OA)_2_:R6G in sodium borate buffer medium (40.69).

Based on the previous results regarding the spectral overlap, the next step consists of a preliminary photophysical study of DEA3HF in the presence of R6G. The concentrations used in this step are shown in Table S2. In this sense, Figure 2c shows the absorption spectra in the borate buffer of the inclusion complex DEA3HF@(OA)_2_ (curve 1), R6G (curve 2), and DEA3HF@(OA)_2_ in the presence of R6G (curve 3). The DEA3HF and R6G presented absorption maxima at 390 nm and 526 nm, respectively. It can be seen that the absorption band at 390 nm of the inclusion complex in the presence of R6G is significantly more intense than the isolated DEA3HF@(OA)_2_, probably due to the interaction of R6G with the OA. In this region (300–450 nm), the contribution of pure rhodamine is practically absent. To confirm the origin of this absorption band, an additional curve was added (dash line), related to the sum of UV spectra from DEA3HF@(OA)_2_ and R6G in sodium tetraborate buffer (curves 1 and 2). It can be observed that even after adding both curves, the signal from the DEA3HF@(OA)_2_:R6G is more intense. These observations lead to the presence of a complex between DEA3HF@(OA)_2_ and R6G. In this particular case, as already mentioned in this study, R6G electrostatically interacts with the exterior of the octa acid, which is consistent with R6G being positively charged and the OA exterior being negatively charged with eight carboxylate anion groups [16], which corroborated with the formation of a complex in the ground state.

The respective fluorescence emission curves are presented in Figure 2d. As already performed in the UV spectra, an additional curve was also added (dash line), related to the sum of spectra from DEA3HF@(OA)_2_ and R6G in ethanol (curves 1 and 2). In this investigation, the main changes are not related to the maxima shift, but to the respective fluorescence intensities. It is worth mentioning that, to avoid comparing solutions with different flavonol and rhodamine concentrations, the stock solution of R6G was previously diluted in the DEA3HF@(OA)_2_ solution, allowing similar concentrations of DEA3HF and R6G in all studied samples. It can be observed that the fluorescence emission spectrum of DEA3HF@(OA)_2_:R6G (curve 3) presented the highest intensity, even when compared to the sum of the individual emissions (dash line), suggesting the existence of an intermolecular interaction between the DEA3HF and the R6G, allowing the formation of an emissive complex or an energy transfer mechanism in the excited state.

**Figure 3:**
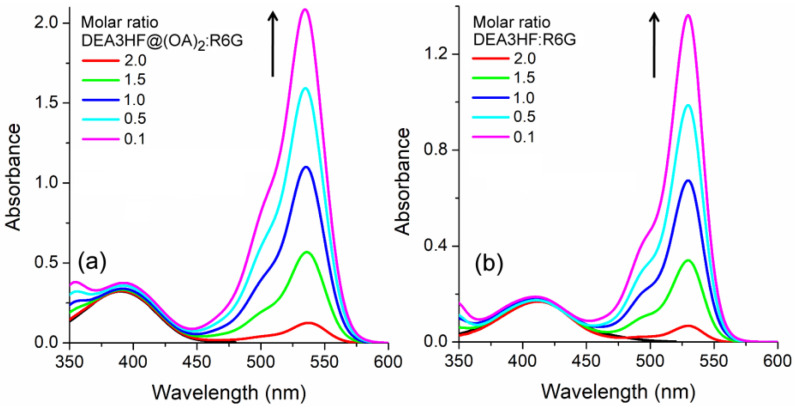
UV-Vis absorption titration for (**a**) DEA3HF@(OA)_2_ in sodium tetraborate buffer and (**b**) DEA3HF in ethanol in the presence of different amounts of R6G. The respective pure flavonol in each system is presented for comparison (black line).

Prompted by these positive observations, the excited state behavior of DEA3HF in solution and included within the deep-cavity cavitand octa acid (OA) in the presence of R6G was explored by steady-state and time-resolved fluorescence emission spectroscopies. In this way, the UV-Vis titration spectra of DEA3HF@(OA)_2_ and DEA3HF in ethanol in the presence of different amounts of R6G are presented in Figure 3a,b, respectively. The titration reveals that the absorption maxima (~390 nm) of DEA3HF in octa-acid and ethanolic solution remain almost unaltered (intensity and position), as expected, while an increase in the intensity of the band located around 530 nm, related to the R6G, is observed.

**Figure 4:**
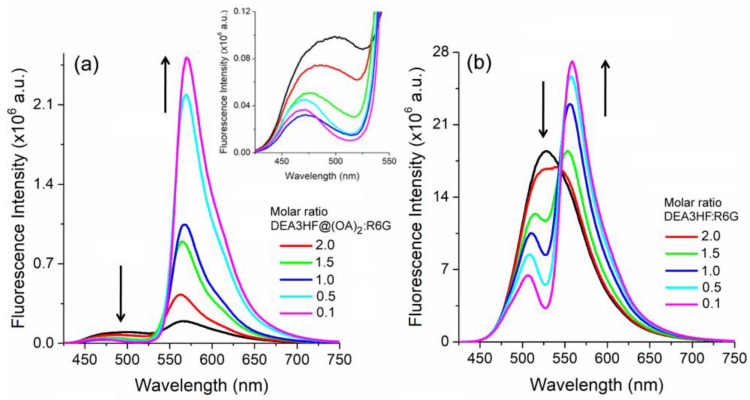
Steady-state fluorescence emission for (**a**) DEA3HF@(OA)_2_ in sodium tetraborate buffer and (**b**) DEA3HF in ethanol in the presence of different amounts of R6G. The insert magnifies the emission from 400–550 nm. The respective pure flavonol in each system is presented for comparison (black line).

The steady-state fluorescence titration spectra of DEA3HF@(OA)_2_ in sodium tetraborate buffer and DEA3HF in ethanol upon the addition of R6G are presented in Figure 4. As can be observed in Figure 4a, in sodium tetraborate buffer medium, after the addition of R6G, the emission at 500 nm related to DEA3HF@(OA)_2_ decreases (Figure 4a, insert), indicating that the HOMO-LUMO recombination in this fluorophore is no longer taking place in the presence of the R6G. It is worth mentioning here that, based on the individual absorption and emission curves from the flavonol and rhodamine (Figure S2), the decrease in fluorescence intensity is not due to a primary inner filter effect, which is sometimes one pitfall for discussion regarding fluorescence quenching [50,51]. In addition, an increase in the emission intensity at 575 nm was also observed. In this region, once again, based on their emission profiles (Figure S2), both DEA3HF and R6G could contribute to the observed signal. However, the emission from free DEA3HF is broader, presenting an FWHM of around 80 nm if compared to the emission from the pure R6G (FWHM~40 nm). Values around 45 nm were calculated at smaller molar ratios (high R6G content), indicating that the increase in the intensity could be related either to the presence of an emissive complex with a rhodamine character or by FRET between the DEA3HF (donor) and the R6G (acceptor). In ethanol, a similar behavior was observed, where when increasing the R6G content, its intensity increases, while the DEA3HF intensity decreases. We would like to highlight that in both sets of experiments, the excitation wavelengths lie with a region where the pure rhodamine does not present absorption (~400 nm). These observations once again indicate that the excited state deactivation involves emission from the complex flavonol:rhodamine or by FRET. It should be noted that the formation of emissive complexes with rhodamines is not a universal trend. In fact, previous studies have reported the formation of a non-emissive RhB-graphene complex through a combination of static and dynamic quenching mechanisms, as revealed by fluorescence quenching studies [52]. Finally, these results are consistent with an interaction between the excited DEA3HF in the inclusion complex, as well as in ethanol solution, and R6G, where the inner filter effect is believed not to be present. 

**Figure 5:**
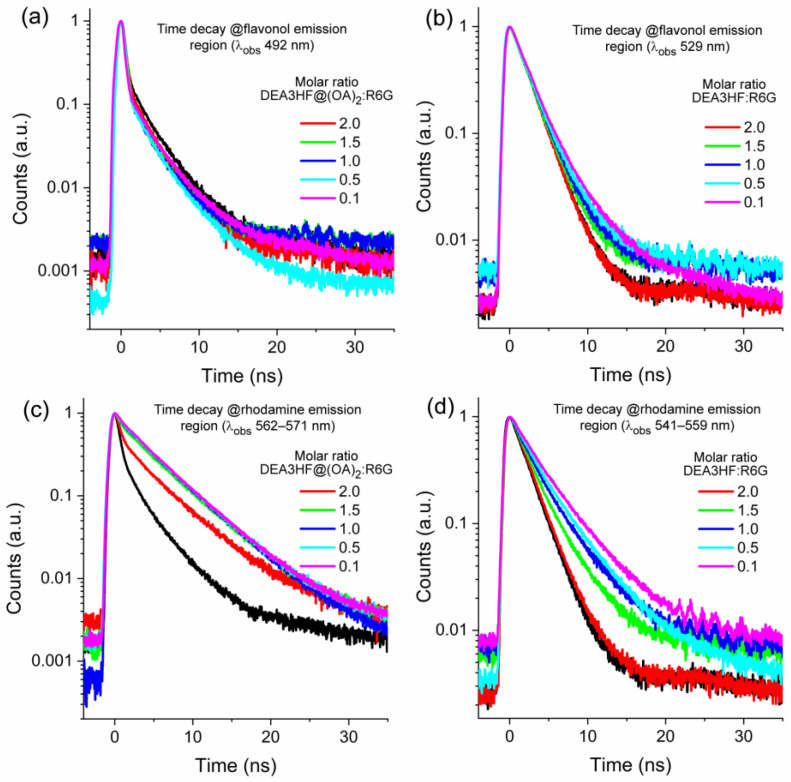
Time-resolved fluorescence emission at two different observation regions (flavonol and rhodamine) for (**a**,**c**) DEA3HF@(OA)_2_ in sodium tetraborate buffer and (**b**,**d**) DEA3HF in ethanol in the presence of different amounts of R6G. The respective pure flavonol in each system is presented for comparison (black line). (Excitation wavelength: 405 nm).

Having gained knowledge concerning the excited state of the studied systems, we carried out fluorescence lifetime measurements to investigate their dynamics in the excited state. This investigation is crucial in the elucidation of the deactivation channel in these systems. For instance, FRET introduces an additional mechanism for dissipating energy from the excited state, by transferring it to its molecular environment, the acceptor. This results in a shorter average lifetime compared to non-FRET systems, due to the increased rate of energy transfer. In this sense, fluorescence lifetime is a useful and direct means of measuring FRET, as it provides valuable insights into the dynamics of energy transfer [38,40]. In this way, Figure 5 presents the titration of DEA3HF@(OA)_2_ in sodium tetraborate buffer and DEA3HF in ethanol in the presence of different amounts of R6G. Since the steady-state results also suggest the presence of an emissive complex, the time decay curves were analyzed at two different emission regions, related to the flavonol and the rhodamine emissions. The relevant data from this investigation are summarized in Table 1 and Table 2 for DEA3HF@(OA)_2_:R6G at both observation wavelengths (flavonol and rhodamine) and in Table 3 and Table 4 for DEA3HF:R6G in ethanol, for the same reason. The original time decay curves, residuals, and the respective complete data are presented as supplementary material (Tables S3–S6, and Figures S6–S29).

The first set of time-resolved curves (Figure 5a,b), related to both systems observed at the DEA3HF emission region, show different results. The system DEA3HF@(OA)_2_:R6G in sodium tetraborate buffer presents an almost constant fluorescence lifetime (~3.0 ns, Table 1), while DEA3HF:R6G in ethanol shows increasing values for the averaged fluorescence lifetime (*τ_av_*) (2.03 up to 2.98 ns) at different amounts of R6G. Despite the diverse behavior between both systems regarding the *τ_av_*, this preliminary result indicates that FRET is no longer expected to be present in their excited states. The inclusion complex DEA3HF@(OA)_2_ presented a two-exponential decay fit (*τ*_1_~1.4 ns and *τ*_2_~3.4 ns, Table 1), as already observed in the literature, where the short lifetime was ascribed to the charge transfer state and the longer one to the ESIPT mechanism, related to the normal (N*) and tautomeric (T*) species, respectively [32]. In addition, the DEA3HF in ethanol showed a mono-exponential time decay (~2.1 ns, Table 3), related to hydrogen-bonded species in this medium, as expected. In the presence of R6G, this behavior is maintained up to a molar ratio of 1.0, where the excited state dynamics are now bi-exponential (*τ*_1_~2.1 ns and *τ*_2_~5.7 ns, Table 3), up to a molar ratio of 0.1 (excess of R6G). At this molar ratio, the two fluorescence lifetimes could be related to the presence of two types of emissive species. The shorter lifetime (*τ*_1_~2.1 ns) is due to hydrogen-bonded DEAFs, as observed at higher molar ratios. However, contrary to that observed for DEA3HF@(OA)_2_:R6G, the longer one (*τ*_2_~5.7 ns), even at a low relative contribution (~20%, Table 3), is probably related to the presence of an emissive complex with an R6G character. This latter observation was based on the magnitude of the fluorescence lifetime of R6G obtained in different environments [53,54].

Since the time-resolved data do not corroborate FRET in these systems, it is also important to investigate their dynamics at the rhodamine emission region (Figure 5c,d), to better visualize the dynamics of the emissive complex. This latter is necessary since the presence of this emissive complex in the ground state is a plausible proposal for the observed fluorescence quenching in both systems. Two-exponential decay fits were observed for DEA3HF@(OA)_2_:R6G in sodium tetraborate buffer (*τ*_1_ ~2.7 ns and *τ*_2_~5.3 ns, Table 2), and for DEA3HF:R6G in ethanol (*τ*_1_~2.3 ns and *τ*_2_~4.6 ns, Table 4). In general, in both systems, the longer lifetime presents the higher contribution (DEA3HF@(OA)_2_:R6G, ~85%—Table 2, and DEA3HF:R6G~55%—Table 4). This latter was expected since the observation regions lie with the rhodamine emission. These preliminary results already indicate that the OA is playing a role in the excited state of the emissive complex since different contributions and time decay values were obtained. In both systems, it is expected that the shorter lifetime is related to DEA3HF@(OA)_2_ or DEA3HF, and the longer ones to the emissive complex formed between DEA3HF@(OA)_2_:R6G or for DEA3HF:R6G.

Since the steady-state and time-resolved results are suggesting the presence of an emissive complex in the ground state, to probe whether the observed fluorescence quenching is a static or dynamic process in these systems, the Stern–Volmer Equation (1) was applied [55,56]:

(1) $$\frac{I_{0}}{I}=1+K_{SV}\left[Q\right]=1+k_{q}\tau_{0}\left[Q\right]$$

where *K_SV_* is the Stern–Volmer quenching constant and indicates the sensitivity of the fluorophore to a quencher, *k_q_* is the bimolecular quenching constant, *τ*_0_ is the unquenched lifetime, and [*Q*] is the quencher concentration. The Stern–Volmer equation can be derived by analyzing the proportion of excited fluorophores that undergo decay by emission, relative to the total number of fluorophores, allowing the Stern–Volmer Equation (2) to be obtained. This equation demonstrates a crucial aspect of collisional quenching, which results in a simultaneous reduction in fluorescence intensity and lifetime (*I*_0_/*I* = *τ*_0_/*τ*). As a consequence, the reduction in the fluorescence quantum yield takes place due to the depopulation of the excited state, without the occurrence of fluorescence emission. In the case of static quenching, the lifetime remains unchanged because only the fluorescent molecules are observed, whereas the uncomplex fluorophores retain their unquenched lifetime *τ*_0_.

(2) $$\frac{\tau_{0}}{\tau }=1+k_{q}\tau_{0}\left[Q\right]$$

**Figure 6:**
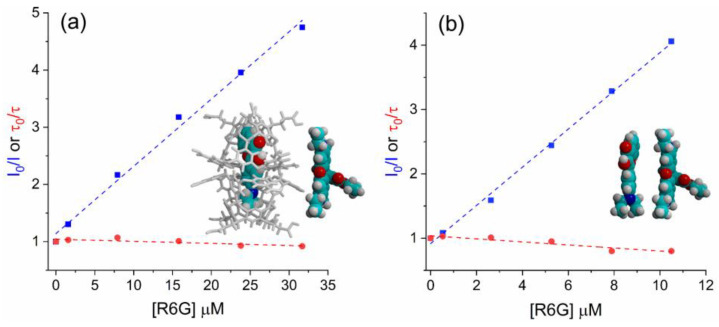
Stern–Volmer plot of (**a**) DEA3HF@(OA)_2_ in sodium tetraborate buffer (R^2^ = 0.993) and (**b**) DEA3HF in ethanol (R^2^ = 0.997) in the presence of different amounts of R6G. The inserts depict a proposal for the respective systems flavonol:rhodamine.

The results of this investigation are presented in Figure 6. The working concentrations are summarized in Table S7. It is worth mentioning that in this calculation, since a bi-exponential time decay was observed in both systems in several molar ratios, an averaged fluorescence lifetime (*τ_av_*) was used (Table 1, Table 2, Table 3 and Table 4). As can be observed in Figure 6, an almost constant *τ_av_*, as well as the *τ*_0_/*τ* ratios close to unity for both sets of DEA3HF:R6G systems, were calculated, suggesting a static mechanism. In addition, a linear fit was obtained for the Stern–Volmer plot (see Figures S30 and S31), which allowed us to obtain the respective association constants *K_SV_* and the bimolecular quenching rate constant (*k_q_*) associated with the quenching efficiency [55]. High *K_SV_* values were obtained (~10^5^ M^−1^), especially for free DEA3HF in ethanol (2.96 × 10^5^ M^−1^) in comparison with DEA3HF@(OA)_2_:R6G (1.18 × 10^5^ M^−1^), indicating moderate to strong interactions between flavonol:rhodamine and flavonol@(OA)_2_:R6G, respectively. Additionally, the *k_q_* values (~10^14^ M^−1^·s^−1^, where DEA3HF@(OA)_2_:R6G = 0.39 × 10^14^ M^−1^·s^−1^ and DEA3HF:R6G = 1.42 × 10^14^ M^−1^·s^−1^) exceed the maximum value for the diffusional collision quenching constant according to the Smoluchowski–Stokes–Einstein theory (*k_diff_* ~7.40 × 10^9^ M^−1^·s^−1^) [56], which indicates that fluorescence quenching occurred by a static mechanism, in which the formation of the paired flavonol:R6G takes place in the ground state.

## 3. Experimental

### 3.1. Materials and Methods

Rhodamine 6G (R6G) was obtained from Aldrich and was used as received. The octa acid was synthesized and purified according to the published procedure [26]. Details from the synthesis and spectroscopic characterization of the DEA3HF, as well as their inclusion complex with octa acid (OA), was already reported in the literature [32]. The complexation of the guest DEA3HF with the host OA was accomplished by vigorously stirring a dimethylsulfoxide solution of the guest in a sodium tetraborate buffer solution (10 mM, pH∼9.0) of the host for a few minutes. The complexation was confirmed by monitoring the ^1^H NMR signals of the host and guest, as already reported in the literature [32], where the guest:host ratio was estimated to be 1:2—DEA3HF:(OA)_2_. Spectroscopic-grade solvents (Aldrich, St. Louis, MO, USA) were used for fluorescence and UV-Visible (UV-Vis) measurements. All experiments were performed at 25 °C. The UV-Vis absorption spectra were performed on a Shimadzu UV-2101PC spectrophotometer (Shimadzu, Kyoto, Japan). Steady-state fluorescence emission spectra were recorded using an Edinburgh Analytical Instruments FS920CDT fluorometer (Edinburgh Analytical Instruments, Livingston, UK). Fluorescence lifetimes were measured by time-correlated single photon counting using an NF920 fluorometer (Edinburgh Instruments). An EPL picosecond pulsed diode laser (EPL405) (Edinburgh Instruments), with a wavelength range of 398–410 nm, and linewidth < 2.0 nm, was used in all time-resolved experiments. The fluorescence decay curves were analyzed using the software F900 (Analysis of Lifetime Data). A nonlinear least square method was employed for the fit of the decay to a sum of exponentials. The intensity averaged fluorescence lifetime (*τ_av_*) was defined and calculated according to Equation (3) [57], where *B_i_* and *τ_i_* are the pre-exponential factors and lifetimes, respectively.

(3) $$\tau_{av}=\frac{\sum_{i=1}^{2}B_{i}\tau_{i}^{2}}{\sum_{i=1}^{2}B_{i}\tau_{i}}$$

### 3.2. Titration Experiments

Stock solutions of DEA3HF@(OA)_2_ (1.56 × 10^−5^ M in borate buffer), DEA3HF (0.52 × 10^−5^ M in anhydrous ethanol), and R6G (1.58 × 10^−3^ M and 4.75 × 10^−3^ M) where prepared for the steady-state and time-resolved fluorescence titration experiments. The more diluted rhodamine solution was used for the titrations with DEA3HF in ethanol, whereas the concentrated rhodamine solution was used for titration with DEA3HF@(OA)_2_. For titration experiments, R6G stock solutions were added stepwise to the DEA3HF solution (DEA3HF@(OA)_2_ or DEA3HF in ethanol). To these measurements, 1.5 mL of DEA3HF stock solution was added in a cuvette and added 0.5, 2.5, 5.0, 7.5, and 10.0 µL of the respective R6G stock solution to obtain different flavonol:rhodamine molar ratios (2.0, 1.5, 1.0, 0.5, and 0.1).

## 4. Conclusions

In summary, this report presents from the perspective of photophysics, which is a detailed spectroscopic analysis of the interaction between a flavonol (DEA3HF) and Rhodamine 6G (R6G) in different environments. In this investigation, the systems DEA3HF closed in an octa acid cavitand and free in ethanolic solution were studied in the presence of R6G. Firstly, in both systems, the principle of spectral overlap was employed, revealing a considerable overlap between the ‘emission’ spectrum of the flavonol and the ‘absorption’ spectrum of the rhodamine. Steady-state photophysics indicated the interaction between the flavonol and the R6G in both proposed systems, where the observed fluorescence quenching could be related due to either the formation of an emissive complex or an energy transfer mechanism in the excited state. Time-resolved fluorescence measurements revealed an almost constant fluorescence lifetime from the DEA3HF upon the addition of R6G, which is not consistent with the FRET mechanism. Finally, from the Stern–Volmer plot, based on the association (*K_SV_*) and the bimolecular quenching rate (*k_q_*) constants, the fluorescence quenching was demonstrated to have a static nature, which corroborates with the formation of an emissive complex in the ground state.

## Figures and Tables

**Table 1:** Time-resolved fluorescence spectroscopy (EPL @405 nm) data from DEA3HF@(OA)_2_ in sodium tetraborate buffer in the presence of different amounts of R6G, where *λ_obs_* is the analyzed emission wavelength, *τ* is the fluorescence lifetime, *τ_av_* is the averaged fluorescence lifetime, Rel. is the relative contribution (%), and χ^2^ is the chi-square of the fit.

Molar Ratio DEA3HF@(OA)_2_:R6G	*λ_obs_* (nm)	*τ*_1_ (ns)	Rel.	*τ*_2_ (ns)	Rel.	χ^2^	*τ_av_* (ns)	*τ*_0_/*τ* ^b^
0 ^a^	492	1.179	16.94	3.320	83.06	1.075	2.96	1.00
2.0	492	1.327	19.20	3.254	80.80	1.080	2.88	1.03
1.5	492	1.292	19.07	3.122	80.93	1.029	2.77	1.07
1.0	492	1.443	22.80	3.367	77.20	1.032	2.93	1.01
0.5	492	1.725	29.71	3.808	70.29	1.015	3.19	0.93
0.1	492	1.570	24.37	3.768	75.63	1.052	3.23	0.92

^a^ DEA3HF@(OA)_2_ in absence of R6G. ^b^ *τ*_0_ is the average fluorescence lifetime in the absence of Rhodamine 6G.

**Table 2:** Time-resolved fluorescence spectroscopy (EPL @405 nm) data from DEA3HF@(OA)_2_ in sodium tetraborate buffer in the presence of different amounts of R6G, where *λ_obs_* is the analyzed emission wavelength, B is the pre-exponential factor, *τ* is the fluorescence lifetime, *τ_av_* is the averaged fluorescence lifetime, Rel. is the relative contribution (%), and χ^2^ is the chi-square of the fit.

Molar Ratio DEA3HF@(OA)_2_:R6G	*λ_obs_* (nm)	*τ*_1_ (ns)	Rel.	*τ*_2_ (ns)	Rel.	χ^2^	*τ_av_* (ns)	*τ*_0_/*τ* ^b^
0 ^a^	566	1.21	21	3.69	79	1.04	3.17	1.00
2.0	562	1.76	11	5.15	89	1.07	4.79	1.51
1.5	564	2.27	6	5.18	94	1.08	5.00	1.58
1.0	567	3.31	24	5.54	76	1.09	5.01	1.58
0.5	569	3.19	17	5.37	83	1.04	4.99	1.58
0.1	571	2.98	16	5.32	84	1.05	4.96	1.56

^a^ DEA3HF@(OA)_2_ in the absence of R6G. ^b^ *τ*_0_ is the average fluorescence lifetime in the absence of Rhodamine 6G.

**Table 3:** Time-resolved fluorescence spectroscopy (EPL @405 nm) data from DEA3HF in ethanol in the presence of different amounts of R6G, where *λ_obs_* is the analyzed emission wavelength, B is the pre-exponential factor, *τ* is the fluorescence lifetime, *τ_av_* is the averaged fluorescence lifetime, Rel. is the relative contribution (%), and χ^2^ is the chi-square of the fit.

Molar Ratio DEA3HF:R6G	*λ_obs_* (nm)	*τ*_1_ (ns)	Rel.	*τ*_2_ (ns)	Rel.	χ^2^	*τ_av_* (ns)	*τ*_0_/*τ* ^b^
0 ^a^	529	2.087	100	-	-	1.101	2.09	1.00
2.0	526	2.030	100	-	-	1.105	2.03	1.03
1.5	529	2.071	100	-	-	1.101	2.07	1.01
1.0	529	2.195	100	-	-	1.118	2.20	0.95
0.5	529	2.097	85.15	5.645	14.85	1.033	2.62	0.80
0.1	529	2.131	76.92	5.789	23.08	1.083	2.98	0.70

^a^ DEA3HF in the absence of R6G. ^b^ *τ*_0_ is the fluorescence lifetime in the absence of Rhodamine 6G.

**Table 4:** Time-resolved fluorescence spectroscopy (EPL @405 nm) data from DEA3HF in ethanol in the presence of different amounts of R6G, where *λ_obs_* is the analyzed emission wavelength, B is the pre-exponential factor, *τ* is the fluorescence lifetime, *τ_av_* is the averaged fluorescence lifetime, Rel. is the relative contribution (%), and χ^2^ is the chi-square of the fit.

Molar Ratio DEA3HF:R6G	*λ_obs_* (nm)	*τ*_1_ (ns)	Rel.	*τ*_2_ (ns)	Rel.	χ^2^	*τ_av_* (ns)	*τ*_0_/*τ* ^b^
0 ^a^	557	2.07	100	-	-	1.08	2.07	1.00
2.0	541	2.14	100	-	-	1.09	2.14	1.03
1.5	553	2.19	67	4.52	33	0.96	2.97	1.43
1.0	556	2.23	44	4.39	56	1.08	3.46	1.67
0.5	557	2.34	35	4.54	65	1.07	3.77	1.82
0.1	559	2.57	32	4.98	68	1.03	4.22	2.04

^a^ DEA3HF in the absence of R6G. ^b^ *τ*_0_ is the fluorescence lifetime in the absence of Rhodamine 6G.

## Data Availability

The data presented in this study are available on request from the corresponding author.

## Supplementary Materials

The following supporting information can be downloaded at: https://www.mdpi.com/article/10.3390/molecules28114260/s1, Figure S1: UV-Vis absorption spectra of R6G in different media; Figure S2: Normalized UV-Vis absorption and steady-state fluorescence emission spectra of (a) DEA3HF@(OA)_2_ in sodium borate buffer, (b) DEA3HF in ethanol, (c) R6G in the presence of DEA3HF@(OA)_2_ in sodium tetraborate buffer, and (d) R6G in ethanol; Figures S3–S29: Time decay curves and respective residuals for the studied systems; Figures S30 and S31: Stern–Volmer plots for the studied systems; Table S1: Relevant data from the time-resolved fluorescence spectroscopy of individual flavonols and rhodamine; Table S2: Working concentrations for the preliminary photophysical study of DEA3HF in the presence of R6G; Table S3–S6: Relevant data from the time-resolved fluorescence spectroscopy for titrations; Table S7: Working concentration for Stern–Volmer plot.
