# Supplementary material for: Spectroscopic Insights of an Emissive Complex between 4′-N,N-Diethylaminoflavonol in Octa-Acid Deep-Cavity Cavitand and Rhodamine 6G
Source: Molecules. 2023 May 23;28(11):4260. doi: 10.3390/molecules28114260 (PMC10254886; doi:10.3390/molecules28114260)
Supplement: Supplementary file 1 [file molecules-28-04260-s001.zip › molecules-2324885-supplementary.pdf]

Supplementary Material

Spectroscopic Insights of an Emissive Complex between 4′-*N*,*N*-Diethylaminoflavonol in Octa-Acid Deep-Cavity Cavitand and Rhodamine 6G

Fabiano da Silveira Santos [1], Elamparuthi Ramasamy [2], Lilian Camargo da Luz [1], Vaidhyanathan Ramamurthy [3,*] and Fabiano Severo Rodembusch [1,*]

[1] Grupo de Pesquisa em Fotoquímica Orgânica Aplicada, Instituto de Química, Universidade Federal do Rio Grande do Sul, Av. Bento Gonçalves 9500, Bairro Agronomia, Porto Alegre CEP 91501-970, Brazil; fabiano@ufrgs.br (F.d.S.S)
[2] Department of Chemistry and Biochemistry, The University of Texas at Arlington, Arlington, TX 76019, USA
[3] Department of Chemistry, University of Miami, 1301 Memorial Drive, Miami, FL 33124, USA
* Correspondence: murthy1@miami.edu (V.R.); Correspondence: rodembusch@iq.ufrgs.br (F.S.R.);
Tel.: +1-305-284-1534 (V.R.); +55-51-3308-9639 (F.S.R.); Fax: +1-305-284-4571 (V.R.); +55-51-3308-7304 (F.S.R.)

Summary

Additional photophysical data 2

Additional photophysical data

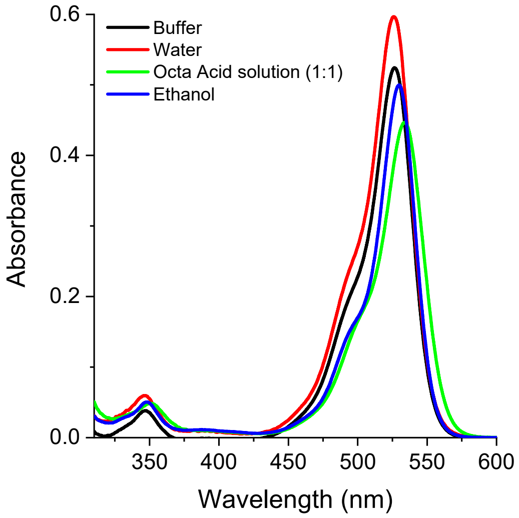

Figure S1. UV-Vis absorption spectra of R6G in different media.

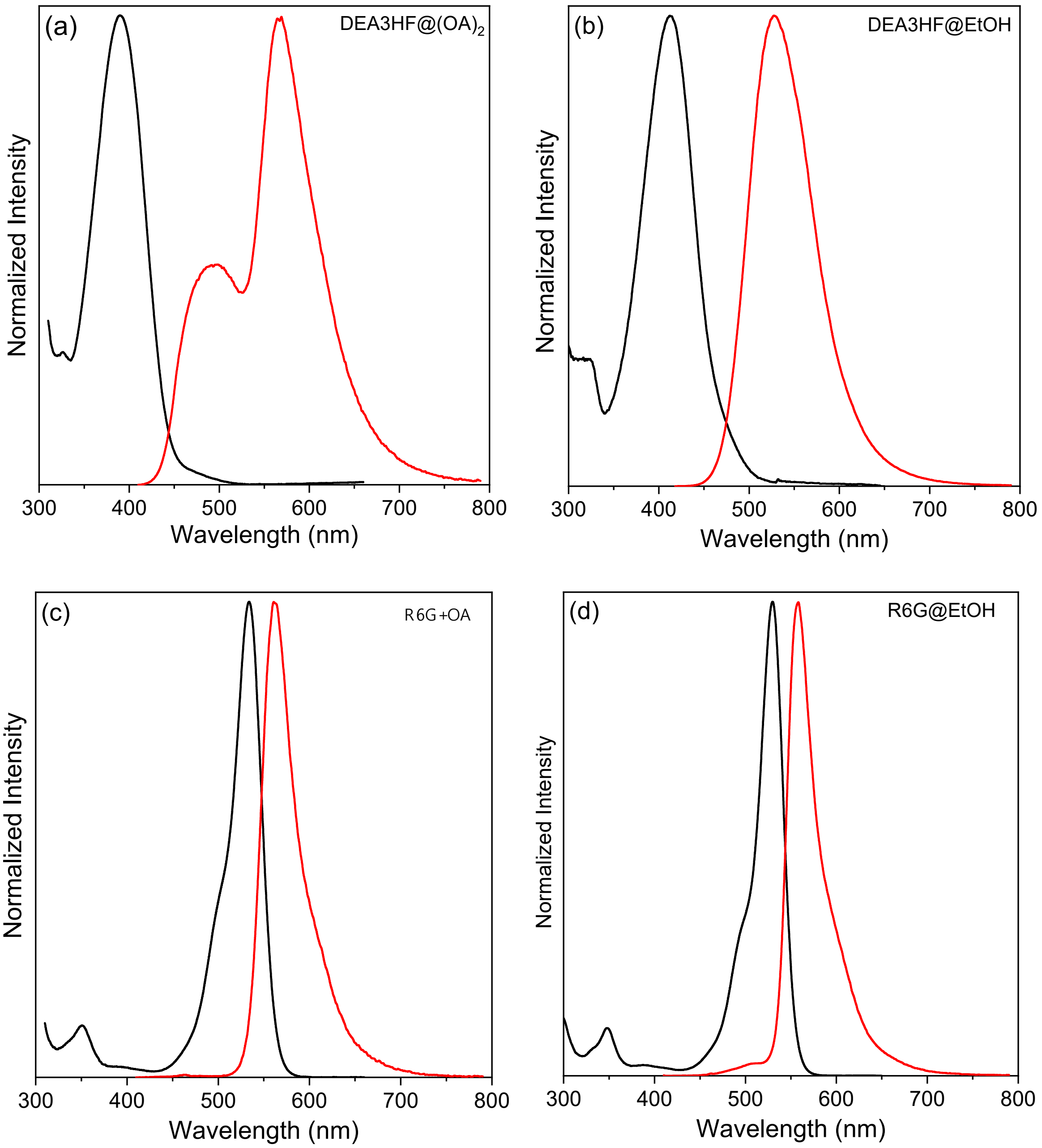

Figure S2. Normalized UV-Vis absorption (black line) and steady-state fluorescence emission (red line) spectra of (a) DEA3HF@$(OA)_2$ in sodium borate buffer, (b) DEA3HF in ethanol, (c) R6G in presence of OA in sodium tetraborate buffer, and (d) R6G in ethanol. The respective emission spectra were acquired using the absorption maxima as excitation wavelength.

Table S1. Relevant data from the time-resolved fluorescence spectroscopy from R6G in different media, where λ_{em} (nm) is the analyzed emission wavelength, B is the pre-exponential factor, τ (ns) is the fluorescence lifetime, Rel. (%) is relative contribution and χ^2 is the chi-square of the fit.

System	Molar Ratio	λ_{em}	B	τ	Rel.	χ^2
R6G in presence of OA	1.0	564	2136.7	4.735	100	1.087
R6G@EtOH	-	557	5998.3	4.387	100	1.075
R6G@Water Buffer	-	556	3563.1	4.256	100	1.104

Excitation Source: EPL405

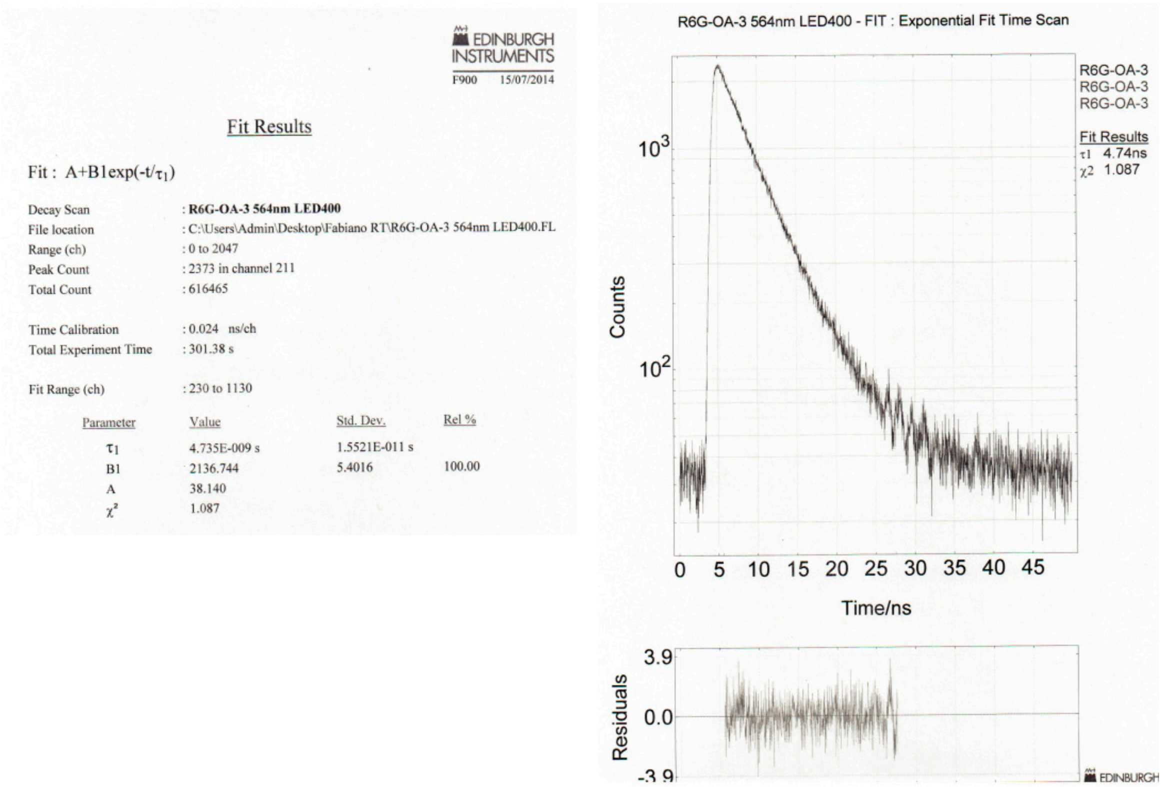

Figure S3. Relevant data from time-resolved fluorescence spectroscopy (left) and time decay curve and respective residuals from [R6G]:[OA]=1.0 (molar ratio). (Excitation wavelength: 405 nm; monitored emission wavelength: 564 nm).

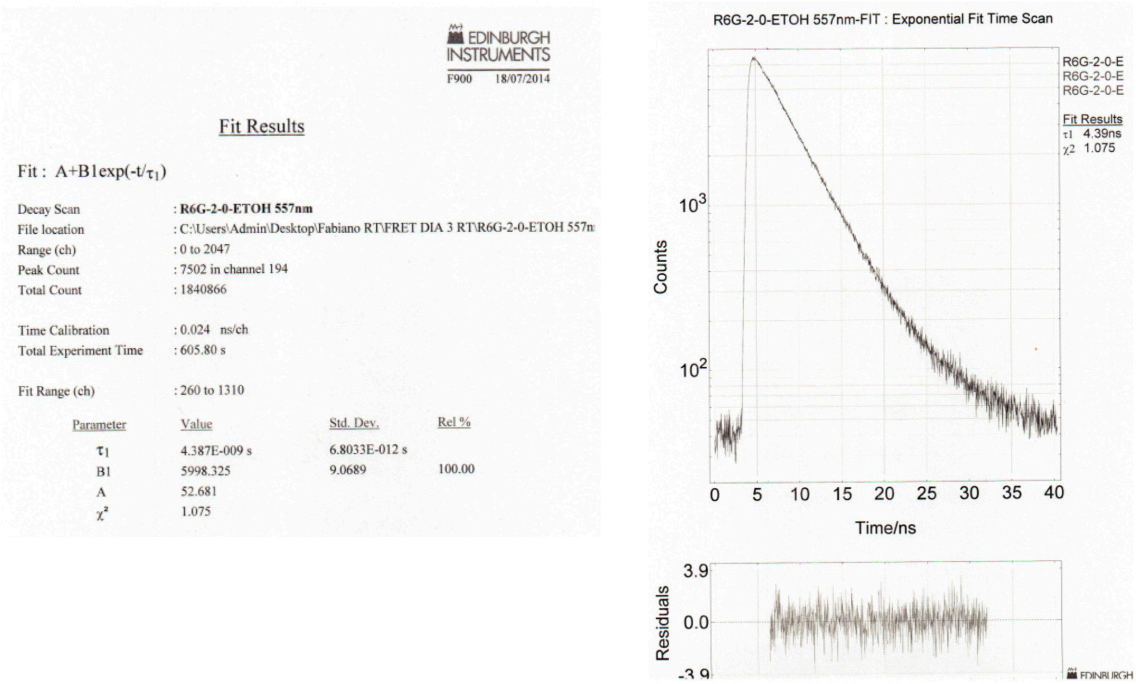

EDINBURGH INSTRUMENTS
F900 18/07/2014

Fit Results

Fit : A+B1exp(-t/τ_1)

Decay Scan	: **R6G-2-0-ETOH 557nm**
File location	: C:\Users\Admin\Desktop\Fabiano RT\FRET DIA 3 RT\R6G-2-0-ETOH 557n
Range (ch)	: 0 to 2047
Peak Count	: 7502 in channel 194
Total Count	: 1840866
Time Calibration	: 0.024 ns/ch
Total Experiment Time	: 605.80 s
Fit Range (ch)	: 260 to 1310

Parameter	Value	Std. Dev.	Rel %
τ_1	4.387E-009 s	6.8033E-012 s	
B1	5998.325	9.0689	100.00
A	52.681		
χ^2	1.075		

Figure S4. Relevant data from time-resolved fluorescence spectroscopy (left) and time decay curve and respective residuals from R6G in ethanol solution. (Excitation wavelength: 405 nm; monitored emission wavelength: 577 nm).

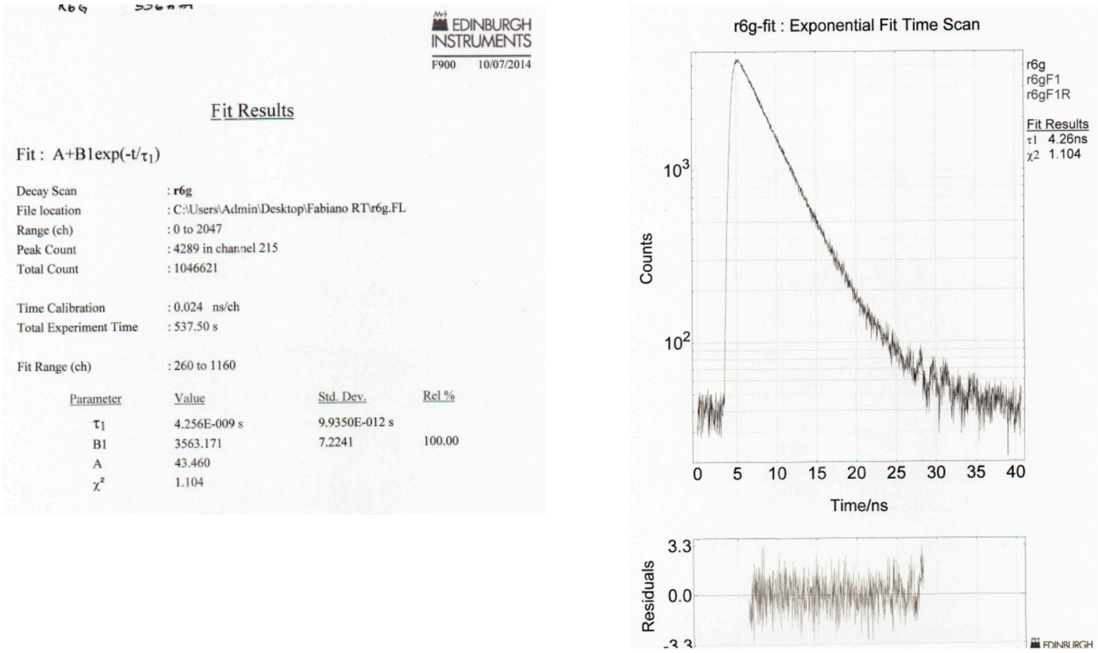

EDINBURGH INSTRUMENTS
F900 10/07/2014

Fit Results

Fit : A+B1exp(-t/τ_1)

Decay Scan	: **r6g**
File location	: C:\Users\Admin\Desktop\Fabiano RT\r6g.FL
Range (ch)	: 0 to 2047
Peak Count	: 4289 in channel 215
Total Count	: 1046621
Time Calibration	: 0.024 ns/ch
Total Experiment Time	: 537.50 s
Fit Range (ch)	: 260 to 1160

Parameter	Value	Std. Dev.	Rel %
τ_1	4.256E-009 s	9.9350E-012 s	
B1	3563.171	7.2241	100.00
A	43.460		
χ^2	1.104		

Figure S5. Relevant data from time-resolved fluorescence spectroscopy (left) and time decay curve and respective residuals from R6G in buffer solution. (Excitation wavelength: 405 nm; monitored emission wavelength: 556 nm).

Table S2. Working concentrations for the photophysical characterization.

Solution	Solvent	Concentration (mol·L^{-1})			
		**DEA3HF (x10^{-5})**	**Octaacid (x10^{-5})**	**Phosphate (x10^{-3})**	**R6G (x10^{-5})**
Buffer	Water	-	-	10	-
Stock 1	Water	1.56	6.24	10	-
Stock 2	Water	-	-	10	1.56
Solution 1	Water	0.78	3.12	10	-
Solution 2	Water	-	-	10	0.78
Solution 3	Water	0.78	3.12	10	0.78

Table S3. Time-resolved fluorescence spectroscopy data from DEA3HF@$(OA)_2$ in sodium tetraborate buffer in the presence of different amounts of R6G, where λ_{obs} is the analyzed emission wavelength, B is the pre-exponential factor, τ is the fluorescence lifetime, Rel. is relative contribution and χ^2 is the chi-square of the fit.

Molar Ratio DEA3HF@$(OA)_2$:R6G	λ_{obs} (nm)	B_1	τ_1 (ns)	Rel. %	B_2	τ_2 (ns)	Rel. %	χ^2	τ_{av} (ns)	τ_0/τ
0	492	958.436	1.179	16.94	1669.098	3.320	83.06	1.075	2.96	1.00
2.0	492	510.165	1.327	19.20	875.271	3.254	80.80	1.080	2.88	1.03
1.5	492	784.478	1.292	19.07	1378.171	3.122	80.93	1.029	2.77	1.07
1.0	492	689.508	1.443	22.80	1000.828	3.367	77.20	1.032	2.93	1.01
0.5	492	1608.641	1.725	29.71	1723.865	3.808	70.29	1.015	3.19	0.93
0.1	492	1837.309	1.570	24.37	2376.601	3.768	75.63	1.052	3.23	0.92

Excitation Source: EPL405

Table S4. Time-resolved fluorescence spectroscopy data from DEA3HF@$(OA)_2$ in sodium tetraborate buffer in the presence of different amounts of R6G, where λ_{obs} is the analyzed emission wavelength, B is the pre-exponential factor, τ is the fluorescence lifetime, Rel. is relative contribution and χ^2 is the chi-square of the fit.

**Molar Ratio DEA3HF@$(OA)_2$:R6G**	λ_{obs} **(nm)**	B_1	τ_1 **(ns)**	**Rel. %**	B_2	τ_2 **(ns)**	**Rel. %**	χ^2	τ_{av} **(ns)**	τ_0/τ
0	566	1980.91	1.21	21	2444.35	3.69	79	1.04	3.17	1.00
2.0	562	893.03	1.76	11	2551.97	5.15	89	1.07	4.79	1.51
1.5	564	1058.11	2.27	6	6859.45	5.18	94	1.08	5.00	1.58
1.0	567	5227.72	3.31	24	9950.59	5.54	76	1.09	5.01	1.58
0.5	569	3734.45	3.19	17	10648.18	5.37	83	1.04	4.99	1.58
0.1	571	5253.29	2.98	16	15957.70	5.32	84	1.05	4.96	1.56

Excitation Source: EPL405

Table S5. Time-resolved fluorescence spectroscopy data from DEA3HF in ethanol in the presence of different amounts of R6G, where λ_{obs} is the analyzed emission wavelength, B is the pre-exponential factor, τ is the fluorescence lifetime, Rel. is relative contribution and χ^2 is the chi-square of the fit.

**Molar Ratio DEA3HF:R6G**	**λ_{obs} (nm)**	**B_1**	**τ_1 (ns)**	**Rel. %**	**B_2**	**τ_2 (ns)**	**Rel. %**	**χ^2**	**τ_{av} (ns)**	**τ_0/τ**
0	529	11341.945	2.087	100	-	-	-	1.101	2.09	1.00
2.0	526	7651.191	2.030	100	-	-	-	1.105	2.03	1.03
1.5	529	9235.787	2.071	100	-	-	-	1.101	2.07	1.01
1.0	529	5526.172	2.195	100	-	-	-	1.118	2.20	0.95
0.5	529	7080.537	2.097	85.15	458.952	5.645	14.85	1.033	2.62	0.80
0.1	529	18793.586	2.131	76.92	2072.220	5.789	23.08	1.083	2.98	0.70

Excitation Source: EPL405

Table S6. Time-resolved fluorescence spectroscopy data from DEA3HF in ethanol in the presence of different amounts of R6G, where λ_{obs} is the analyzed emission wavelength, B is the pre-exponential factor, τ is the fluorescence lifetime, Rel. is relative contribution and χ^2 is the chi-square of the fit.

**Molar Ratio DEA3HF@(OA)$_2$:R6G**	**λ_{obs} (nm)**	**B_1**	**τ_1 (ns)**	**Rel. %**	**B_2**	**τ_2 (ns)**	**Rel. %**	**χ^2**	**τ_{av} (ns)**	**τ_0/τ**
0	557	7469.79	2.07	100	-	-	-	1.08	2.07	1.00
2.0	541	14030.72	2.14	100	-	-	-	1.09	2.14	1.03
1.5	553	7371.43	2.19	67	1798.74	4.52	33	0.96	2.97	1.43
1.0	556	4607.63	2.23	44	3079.30	4.39	56	1.08	3.46	1.67
0.5	557	6537.20	2.34	35	6206.55	4.54	65	1.07	3.77	1.82
0.1	559	4871.56	2.57	32	5442.68	4.98	68	1.03	4.22	2.04

Excitation Source: EPL405

ZERO 492 nm

EDINBURGH INSTRUMENTS
F900 15/07/2014

Fit Results

Fit : A+B1exp(-t/τ_1)+B2exp(-t/τ_2)

Decay Scan	: **Decay21**
File location	: File has not been saved.
Range (ch)	: 0 to 2047
Peak Count	: 15544 in channel 176
Total Count	: 1072799
Time Calibration	: 0.024 ns/ch
Total Experiment Time	: 465.75 s
Fit Range (ch)	: 230 to 945

Parameter	Value	Std. Dev.	Rel %
τ_1	1.179E-009 s	6.7689E-011 s	
τ_2	3.320E-009 s	5.8057E-011 s	
B1	958.436	57.3605	16.94
B2	1669.098	62.1076	83.06
A	30.443		
χ^2	1.075		

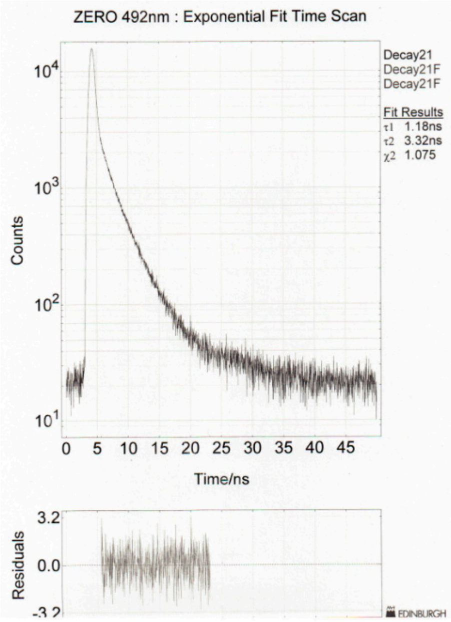

Figure S6. Relevant data from time-resolved fluorescence spectroscopy (left) and time decay curve and respective residuals from DEA3HF@$(OA)_2$ in sodium tetraborate buffer. (Excitation wavelength: 405 nm; monitored emission wavelength: 492 nm).

EDINBURGH INSTRUMENTS
F900 15/07/2014

Fit Results

Fit : A+B1exp(-t/τ_1)+B2exp(-t/τ_2)

Decay Scan	: **0-1 492nm**
File location	: C:\Users\Admin\Desktop\Fabiano RT\0-1 492nm.FL
Range (ch)	: 0 to 2047
Peak Count	: 12366 in channel 174
Total Count	: 798933
Time Calibration	: 0.024 ns/ch
Total Experiment Time	: 407.10 s
Fit Range (ch)	: 247 to 876

Parameter	Value	Std. Dev.	Rel %
τ_1	1.327E-009 s	1.4296E-010 s	
τ_2	3.254E-009 s	1.3676E-010 s	
B1	510.165	80.4547	19.20
B2	875.271	84.1646	80.80
A	19.117		
χ^2	1.088		

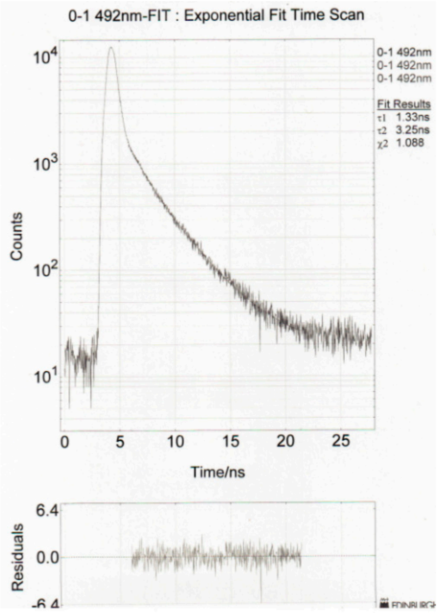

Figure S7. Relevant data from time-resolved fluorescence spectroscopy (left) and time decay curve and respective residuals from DEA3HF@$(OA)_2$:R6G molar ratio=2.0 in sodium tetraborate buffer. (Excitation wavelength: 405 nm; monitored emission wavelength: 492 nm).

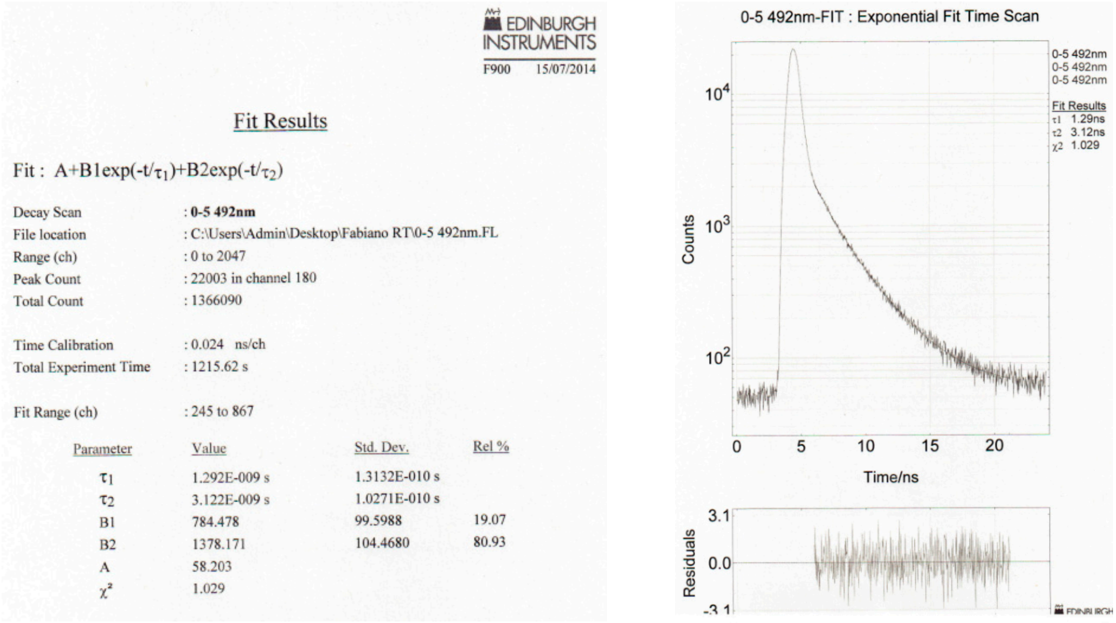

EDINBURGH INSTRUMENTS
F900 15/07/2014

Fit Results

Fit : $A+B1\exp(-t/\tau_1)+B2\exp(-t/\tau_2)$

Decay Scan : **0-5 492nm**
File location : C:\Users\Admin\Desktop\Fabiano RT\0-5 492nm.FL
Range (ch) : 0 to 2047
Peak Count : 22003 in channel 180
Total Count : 1366090

Time Calibration : 0.024 ns/ch
Total Experiment Time : 1215.62 s

Fit Range (ch) : 245 to 867

Parameter	Value	Std. Dev.	Rel %
τ_1	1.292E-009 s	1.3132E-010 s	
τ_2	3.122E-009 s	1.0271E-010 s	
B1	784.478	99.5988	19.07
B2	1378.171	104.4680	80.93
A	58.203		
χ^2	1.029		

Figure S8. Relevant data from time-resolved fluorescence spectroscopy (left) and time decay curve and respective residuals from DEA3HF@(OA)$_2$:R6G molar ratio=1.5 in sodium tetraborate buffer @492 nm. (Excitation wavelength: 405 nm; monitored emission wavelength: 492 nm).

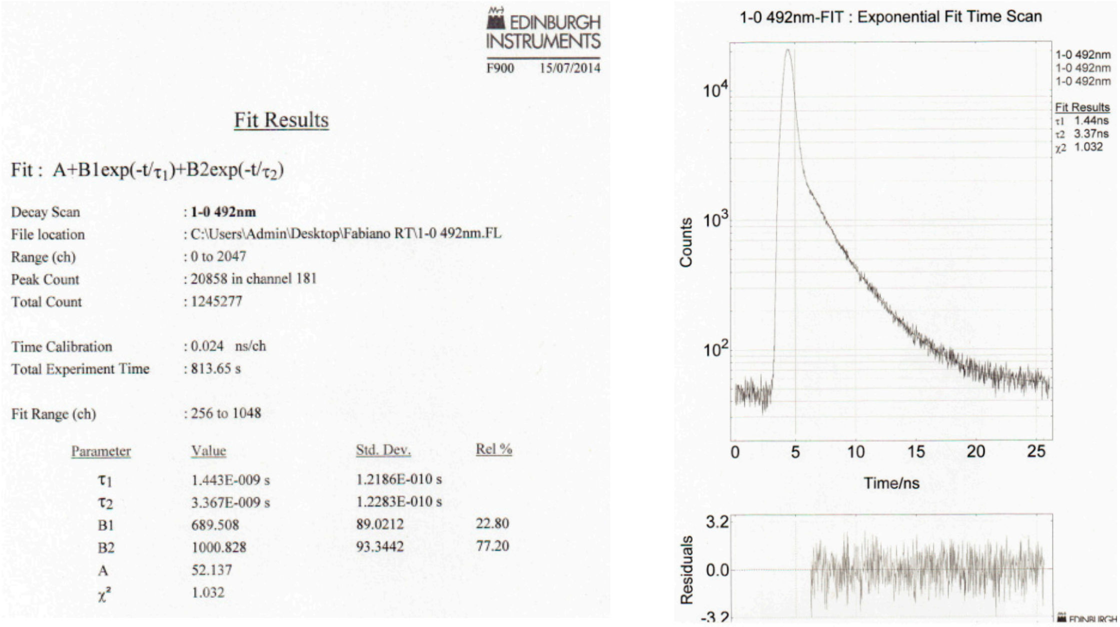

EDINBURGH INSTRUMENTS
F900 15/07/2014

Fit Results

Fit : $A+B1\exp(-t/\tau_1)+B2\exp(-t/\tau_2)$

Decay Scan : **1-0 492nm**
File location : C:\Users\Admin\Desktop\Fabiano RT\1-0 492nm.FL
Range (ch) : 0 to 2047
Peak Count : 20858 in channel 181
Total Count : 1245277

Time Calibration : 0.024 ns/ch
Total Experiment Time : 813.65 s

Fit Range (ch) : 256 to 1048

Parameter	Value	Std. Dev.	Rel %
τ_1	1.443E-009 s	1.2186E-010 s	
τ_2	3.367E-009 s	1.2283E-010 s	
B1	689.508	89.0212	22.80
B2	1000.828	93.3442	77.20
A	52.137		
χ^2	1.032		

Figure S9. Relevant data from time-resolved fluorescence spectroscopy (left) and time decay curve and respective residuals from DEA3HF@(OA)$_2$:R6G molar ratio=1.0 in sodium tetraborate buffer. (Excitation wavelength: 405 nm; monitored emission wavelength: 492 nm).

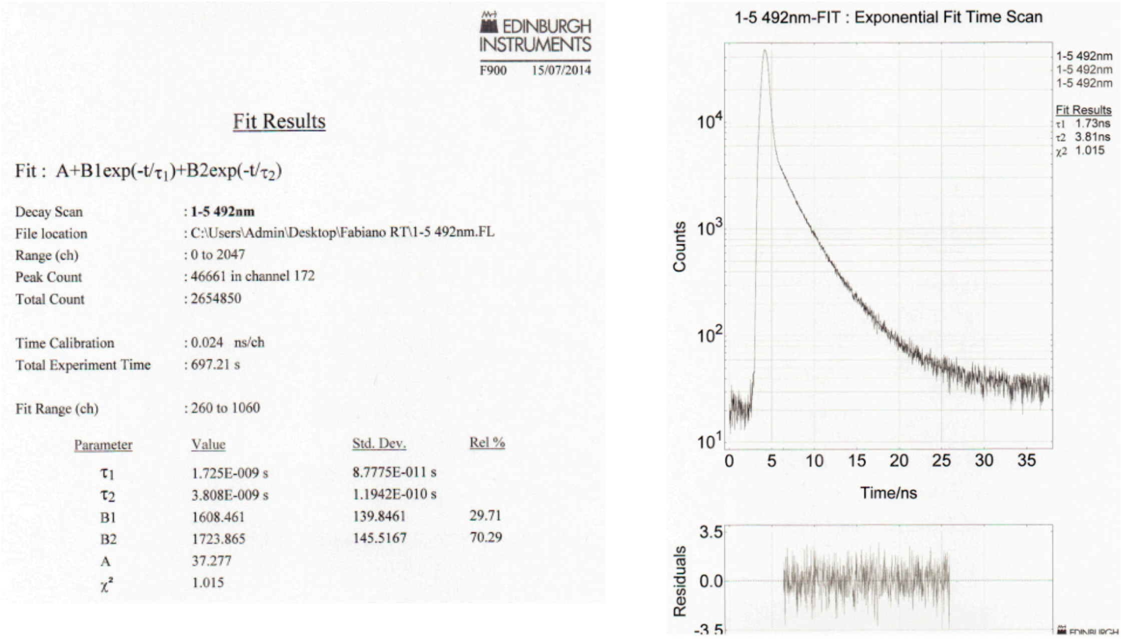

Figure S10. Relevant data from time-resolved fluorescence spectroscopy (left) and time decay curve and respective residuals from DEA3HF@$(OA)_2$:R6G molar ratio=0.5 in sodium tetraborate buffer. (Excitation wavelength: 405 nm; monitored emission wavelength: 492 nm).

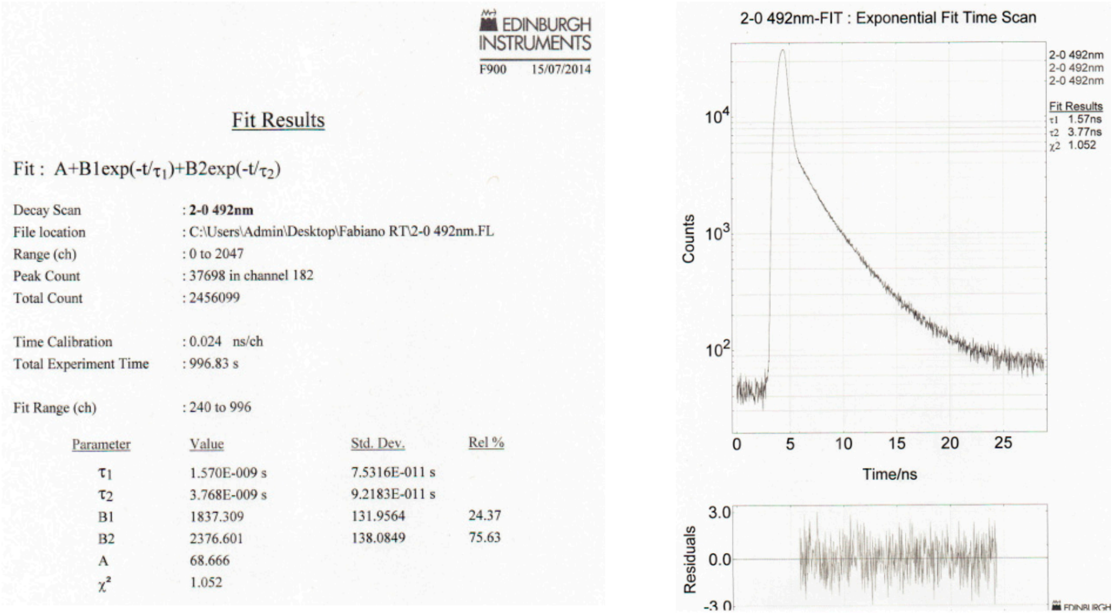

Figure S11. Relevant data from time-resolved fluorescence spectroscopy (left) and time decay curve and respective residuals from DEA3HF@$(OA)_2$:R6G molar ratio=0.1 in sodium tetraborate buffer. (Excitation wavelength: 405 nm; monitored emission wavelength: 492 nm).

EDINBURGH INSTRUMENTS

F900 15/07/2014

Fit Results

Fit : A+B1exp(-t/τ_1)+B2exp(-t/τ_2)

Decay Scan	: **ZERO 566nm MEDIDA 2**
File location	: C:\Users\Admin\Desktop\Fabiano RT\ZERO 566nm MEDIDA 2.FL
Range (ch)	: 0 to 2047
Peak Count	: 18599 in channel 186
Total Count	: 1597273
Time Calibration	: 0.024 ns/ch
Total Experiment Time	: 465.26 s
Fit Range (ch)	: 245 to 994

Parameter	Value	Std. Dev.	Rel %
τ_1	1.211E-009 s	3.8414E-011 s	
τ_2	3.691E-009 s	4.9410E-011 s	
B1	1980.918	58.0146	21.00
B2	2444.353	63.1960	79.00
A	45.851		
χ^2	1.036		

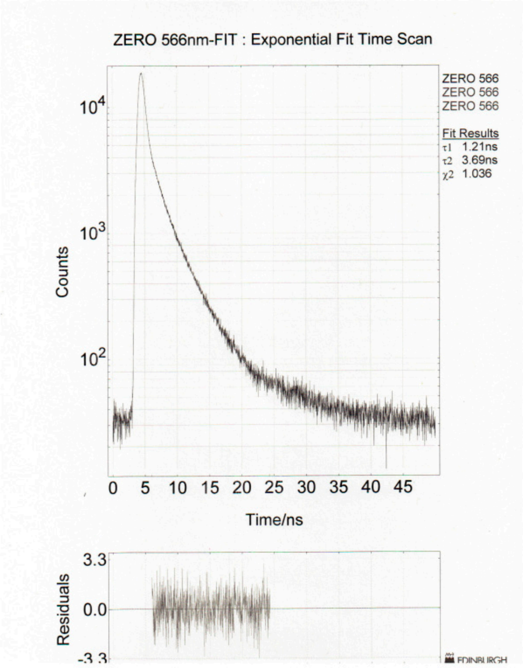

Figure S12. Relevant data from time-resolved fluorescence spectroscopy (left) and time decay curve and respective residuals from DEA3HF@$(OA)_2$ in sodium tetraborate buffer. (Excitation wavelength: 405 nm; monitored emission wavelength: 566 nm).

EDINBURGH INSTRUMENTS

F900 15/07/2014

Fit Results

Fit : A+B1exp(-t/τ_1)+B2exp(-t/τ_2)

Decay Scan	: **0-1 562NM**
File location	: C:\Users\Admin\Desktop\Fabiano RT\0-1 562NM.FL
Range (ch)	: 0 to 2047
Peak Count	: 7593 in channel 186
Total Count	: 1139223
Time Calibration	: 0.024 ns/ch
Total Experiment Time	: 443.80 s
Fit Range (ch)	: 240 to 1380

Parameter	Value	Std. Dev.	Rel %
τ_1	1.766E-009 s	9.5848E-011 s	
τ_2	5.151E-009 s	4.8631E-011 s	
B1	893.036	46.8362	10.71
B2	2551.972	50.9041	89.29
A	30.136		
χ^2	1.071		

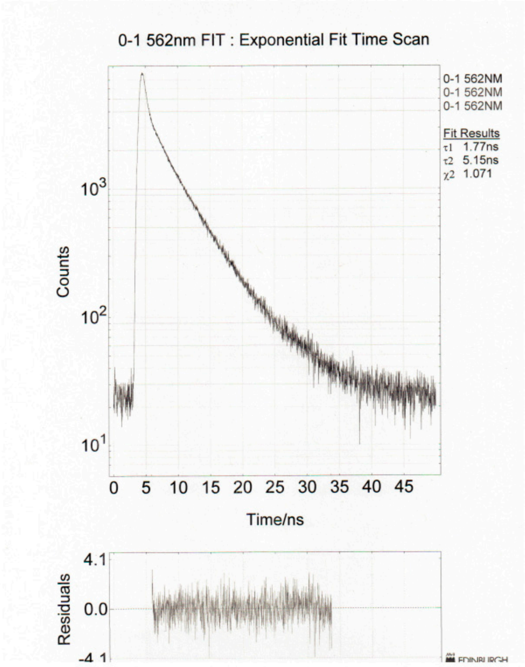

Figure S13. Relevant data from time-resolved fluorescence spectroscopy (left) and time decay curve and respective residuals from DEA3HF@$(OA)_2$:R6G molar ratio=2.0 in sodium tetraborate buffer. (Excitation wavelength: 405 nm; monitored emission wavelength: 562 nm).

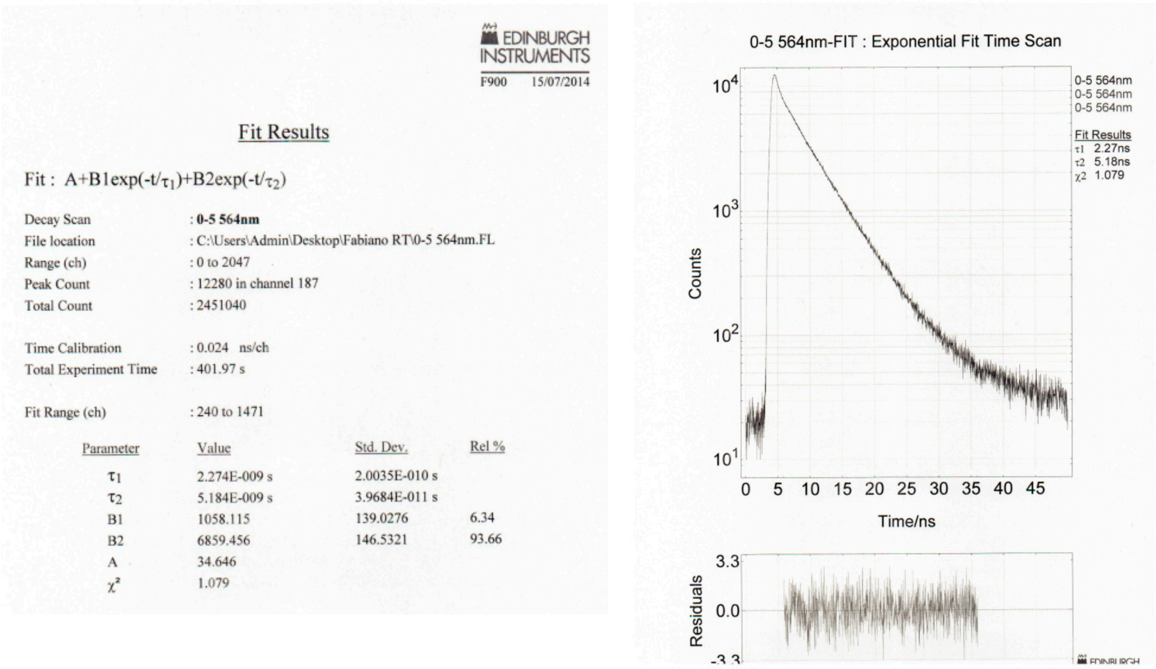

EDINBURGH INSTRUMENTS
F900 15/07/2014

Fit Results

Fit : A+B1exp(-t/τ_1)+B2exp(-t/τ_2)

Decay Scan	: **0-5 564nm**
File location	: C:\Users\Admin\Desktop\Fabiano RT\0-5 564nm.FL
Range (ch)	: 0 to 2047
Peak Count	: 12280 in channel 187
Total Count	: 2451040
Time Calibration	: 0.024 ns/ch
Total Experiment Time	: 401.97 s
Fit Range (ch)	: 240 to 1471

Parameter	Value	Std. Dev.	Rel %
τ_1	2.274E-009 s	2.0035E-010 s	
τ_2	5.184E-009 s	3.9684E-011 s	
B1	1058.115	139.0276	6.34
B2	6859.456	146.5321	93.66
A	34.646		
χ^2	1.079		

Figure S14. Relevant data from time-resolved fluorescence spectroscopy (left) and time decay curve and respective residuals from DEA3HF@$(OA)_2$:R6G molar ratio=1.5 in sodium tetraborate buffer. (Excitation wavelength: 405 nm; monitored emission wavelength: 564 nm).

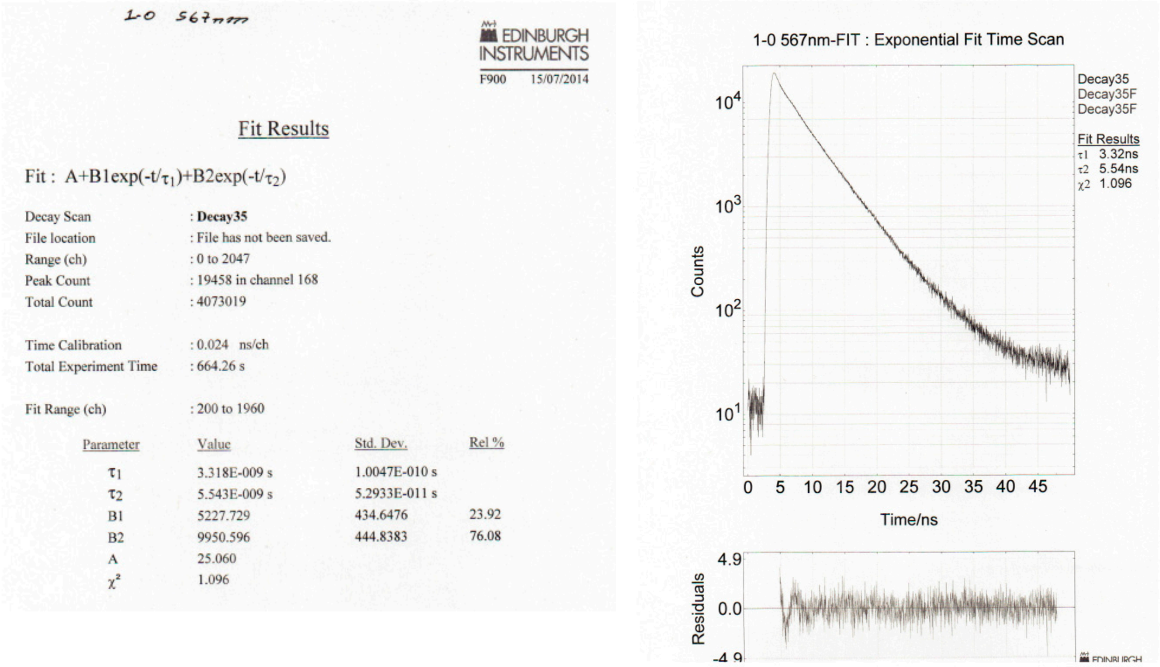

1-0 567nm

EDINBURGH INSTRUMENTS
F900 15/07/2014

Fit Results

Fit : A+B1exp(-t/τ_1)+B2exp(-t/τ_2)

Decay Scan	: **Decay35**
File location	: File has not been saved.
Range (ch)	: 0 to 2047
Peak Count	: 19458 in channel 168
Total Count	: 4073019
Time Calibration	: 0.024 ns/ch
Total Experiment Time	: 664.26 s
Fit Range (ch)	: 200 to 1960

Parameter	Value	Std. Dev.	Rel %
τ_1	3.318E-009 s	1.0047E-010 s	
τ_2	5.543E-009 s	5.2933E-011 s	
B1	5227.729	434.6476	23.92
B2	9950.596	444.8383	76.08
A	25.060		
χ^2	1.096		

Figure S15. Relevant data from time-resolved fluorescence spectroscopy (left) and time decay curve and respective residuals from DEA3HF@$(OA)_2$:R6G molar ratio=1.0 in sodium tetraborate buffer. (Excitation wavelength: 405 nm; monitored emission wavelength: 567 nm).

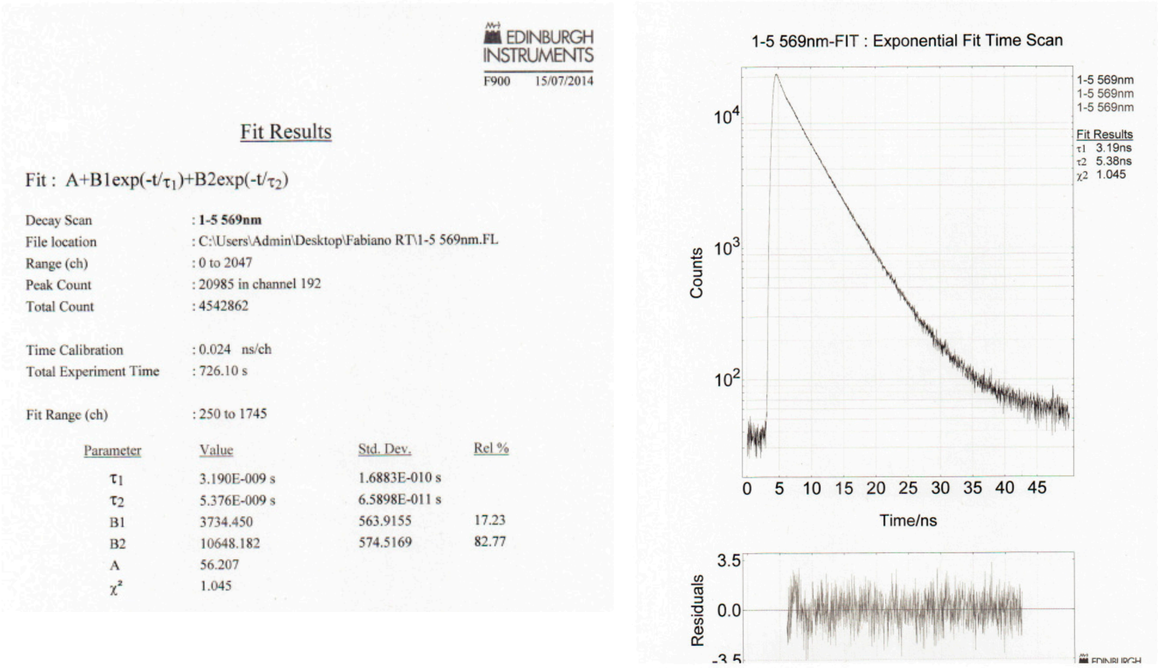

EDINBURGH INSTRUMENTS
F900 15/07/2014

Fit Results

Fit : A+B1exp(-t/τ_1)+B2exp(-t/τ_2)

Decay Scan	: 1-5 569nm
File location	: C:\Users\Admin\Desktop\Fabiano RT\1-5 569nm.FL
Range (ch)	: 0 to 2047
Peak Count	: 20985 in channel 192
Total Count	: 4542862
Time Calibration	: 0.024 ns/ch
Total Experiment Time	: 726.10 s
Fit Range (ch)	: 250 to 1745

Parameter	Value	Std. Dev.	Rel %
τ_1	3.190E-009 s	1.6883E-010 s	
τ_2	5.376E-009 s	6.5898E-011 s	
B1	3734.450	563.9155	17.23
B2	10648.182	574.5169	82.77
A	56.207		
χ^2	1.045		

Figure S16. Relevant data from time-resolved fluorescence spectroscopy (left) and time decay curve and respective residuals from DEA3HF@(OA)$_2$:R6G molar ratio=0.5 in sodium tetraborate buffer. (Excitation wavelength: 405 nm; monitored emission wavelength: 569 nm).

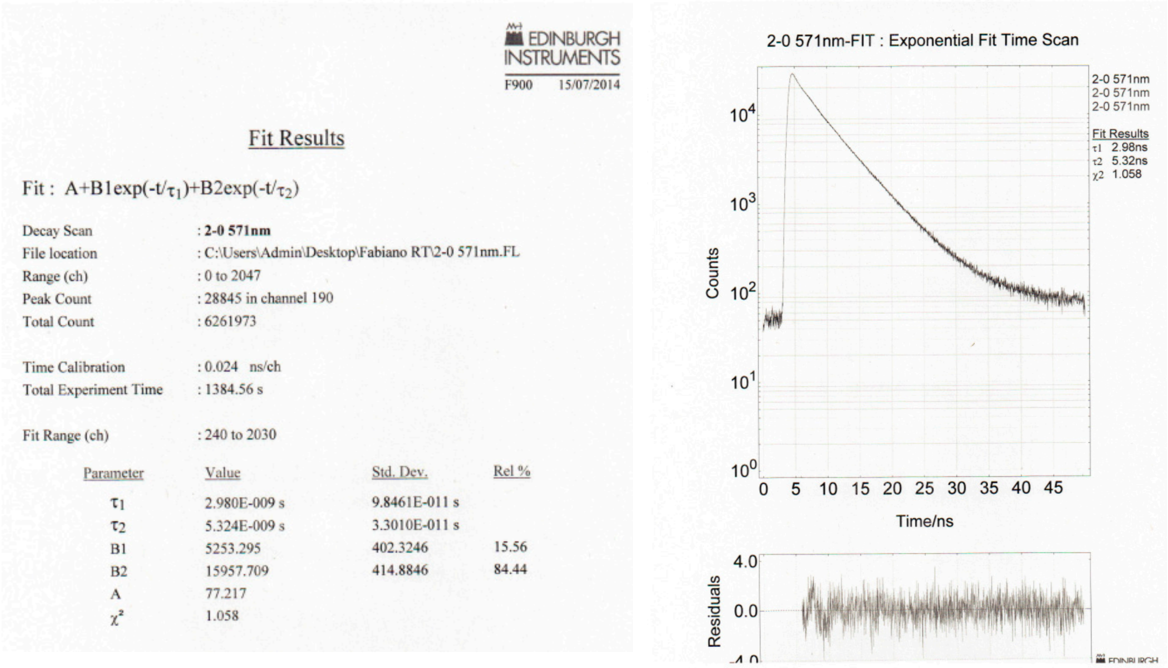

EDINBURGH INSTRUMENTS
F900 15/07/2014

Fit Results

Fit : A+B1exp(-t/τ_1)+B2exp(-t/τ_2)

Decay Scan	: 2-0 571nm
File location	: C:\Users\Admin\Desktop\Fabiano RT\2-0 571nm.FL
Range (ch)	: 0 to 2047
Peak Count	: 28845 in channel 190
Total Count	: 6261973
Time Calibration	: 0.024 ns/ch
Total Experiment Time	: 1384.56 s
Fit Range (ch)	: 240 to 2030

Parameter	Value	Std. Dev.	Rel %
τ_1	2.980E-009 s	9.8461E-011 s	
τ_2	5.324E-009 s	3.3010E-011 s	
B1	5253.295	402.3246	15.56
B2	15957.709	414.8846	84.44
A	77.217		
χ^2	1.058		

Figure S17. Relevant data from time-resolved fluorescence spectroscopy (left) and time decay curve and respective residuals from DEA3HF@(OA)$_2$:R6G molar ratio=0.1 in sodium tetraborate buffer. (Excitation wavelength: 405 nm; monitored emission wavelength: 571 nm).

EDINBURGH INSTRUMENTS
F900 18/07/2014

Fit Results

Fit : A+B1exp(-t/τ_1)

Decay Scan	: **ZERO 529nm**
File location	: C:\Users\Admin\Desktop\Fabiano RT\FRET DIA 3 RT\ZERO 529nm.FL
Range (ch)	: 0 to 2047
Peak Count	: 13756 in channel 194
Total Count	: 1772411
Time Calibration	: 0.024 ns/ch
Total Experiment Time	: 622.27 s
Fit Range (ch)	: 220 to 734

Parameter	Value	Std. Dev.	Rel %
τ_1	2.087E-009 s	3.1653E-012 s	
B1	11341.945	17.8535	100.00
A	44.595		
χ^2	1.101		

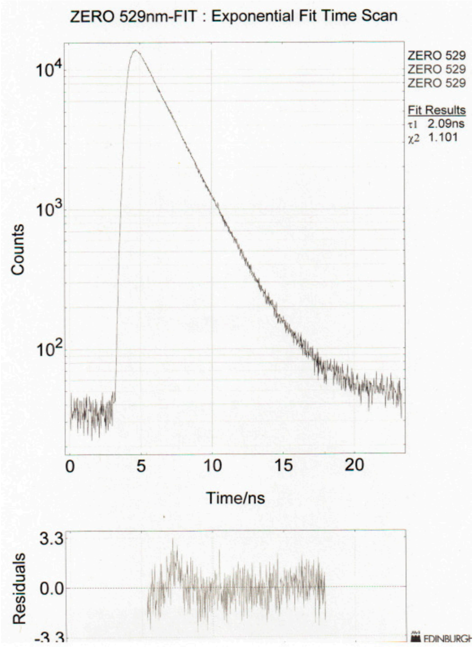

Figure S18. Relevant data from time-resolved fluorescence spectroscopy (left) and time decay curve and respective residuals from DEA3HF in ethanol. (Excitation wavelength: 405 nm; monitored emission wavelength: 529 nm).

EDINBURGH INSTRUMENTS
F900 18/07/2014

Fit Results

Fit : A+B1exp(-t/τ_1)

Decay Scan	: **0-1 526nm**
File location	: C:\Users\Admin\Desktop\Fabiano RT\FRET DIA 3 RT\0-1 526nm.FL
Range (ch)	: 0 to 2047
Peak Count	: 19037 in channel 192
Total Count	: 2399716
Time Calibration	: 0.024 ns/ch
Total Experiment Time	: 741.70 s
Fit Range (ch)	: 280 to 820

Parameter	Value	Std. Dev.	Rel %
τ_1	2.030E-009 s	3.8109E-012 s	
B1	7651.191	14.9793	100.00
A	65.294		
χ^2	1.105		

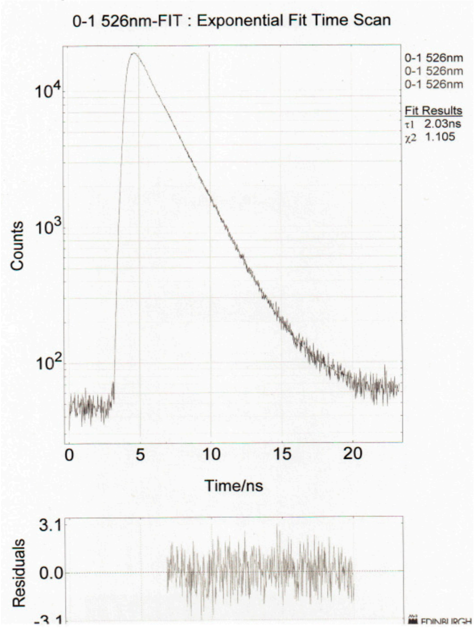

Figure S19. Relevant data from time-resolved fluorescence spectroscopy (left) and time decay curve and respective residuals from DEA3HF:R6G molar ratio=2.0 in ethanol. (Excitation wavelength: 405 nm; monitored emission wavelength: 526 nm).

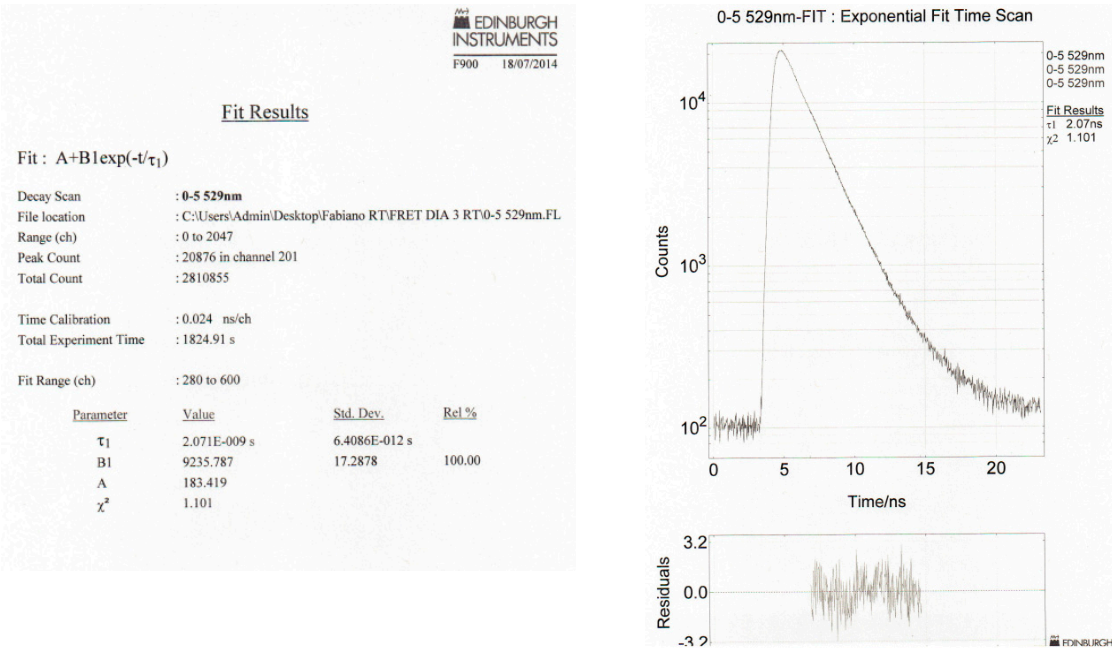
EDINBURGH INSTRUMENTS
F900 18/07/2014

Fit Results

Fit : A+B1exp(-t/τ_1)

Decay Scan	: **0-5 529nm**
File location	: C:\Users\Admin\Desktop\Fabiano RT\FRET DIA 3 RT\0-5 529nm.FL
Range (ch)	: 0 to 2047
Peak Count	: 20876 in channel 201
Total Count	: 2810855
Time Calibration	: 0.024 ns/ch
Total Experiment Time	: 1824.91 s
Fit Range (ch)	: 280 to 600

Parameter	Value	Std. Dev.	Rel %
τ_1	2.071E-009 s	6.4086E-012 s	
B1	9235.787	17.2878	100.00
A	183.419		
χ^2	1.101		

Figure S20. Relevant data from time-resolved fluorescence spectroscopy (left) and time decay curve and respective residuals from DEA3HF:R6G molar ratio=1.5 in ethanol. (Excitation wavelength: 405 nm; monitored emission wavelength: 529 nm).

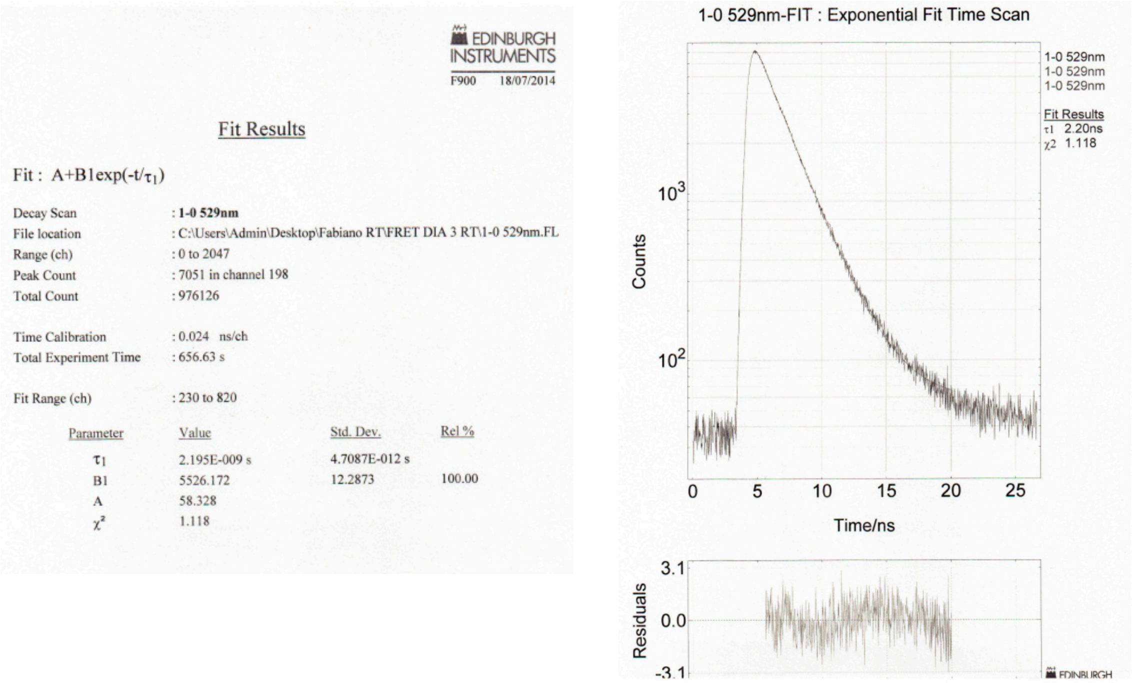
EDINBURGH INSTRUMENTS
F900 18/07/2014

Fit Results

Fit : A+B1exp(-t/τ_1)

Decay Scan	: **1-0 529nm**
File location	: C:\Users\Admin\Desktop\Fabiano RT\FRET DIA 3 RT\1-0 529nm.FL
Range (ch)	: 0 to 2047
Peak Count	: 7051 in channel 198
Total Count	: 976126
Time Calibration	: 0.024 ns/ch
Total Experiment Time	: 656.63 s
Fit Range (ch)	: 230 to 820

Parameter	Value	Std. Dev.	Rel %
τ_1	2.195E-009 s	4.7087E-012 s	
B1	5526.172	12.2873	100.00
A	58.328		
χ^2	1.118		

Figure S21. Relevant data from time-resolved fluorescence spectroscopy (left) and time decay curve and respective residuals from DEA3HF:R6G molar ratio=1.0 in ethanol. (Excitation wavelength: 405 nm; monitored emission wavelength: 529 nm).

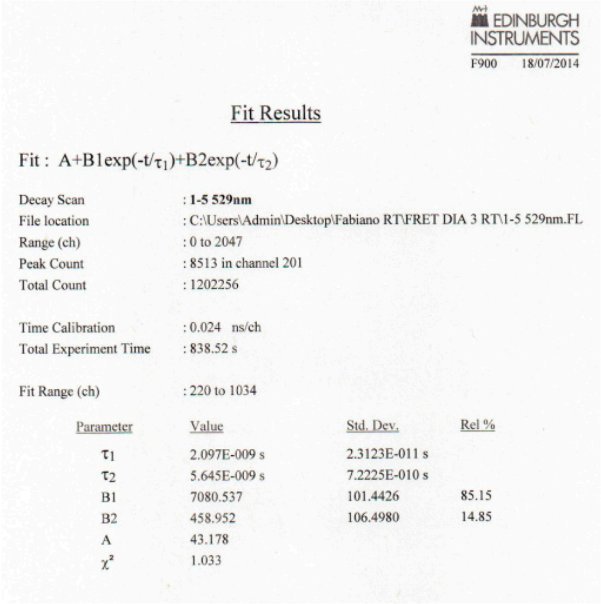

EDINBURGH INSTRUMENTS

F900 18/07/2014

Fit Results

Fit : $A+B1\exp(-t/\tau_1)+B2\exp(-t/\tau_2)$

Decay Scan	: 1-5 529nm
File location	: C:\Users\Admin\Desktop\Fabiano RT\FRET DIA 3 RT\1-5 529nm.FL
Range (ch)	: 0 to 2047
Peak Count	: 8513 in channel 201
Total Count	: 1202256
Time Calibration	: 0.024 ns/ch
Total Experiment Time	: 838.52 s
Fit Range (ch)	: 220 to 1034

Parameter	Value	Std. Dev.	Rel %
τ_1	2.097E-009 s	2.3123E-011 s	
τ_2	5.645E-009 s	7.2225E-010 s	
B1	7080.537	101.4426	85.15
B2	458.952	106.4980	14.85
A	43.178		
χ^2	1.033		

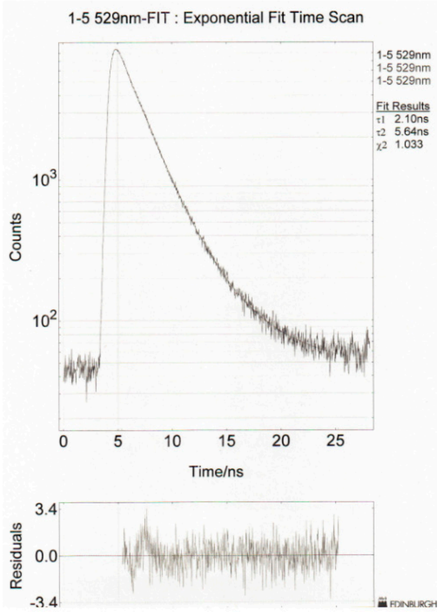

Figure S22. Relevant data from time-resolved fluorescence spectroscopy (left) and time decay curve and respective residuals from DEA3HF:R6G molar ratio=0.5 in ethanol. (Excitation wavelength: 405 nm; monitored emission wavelength: 529 nm).

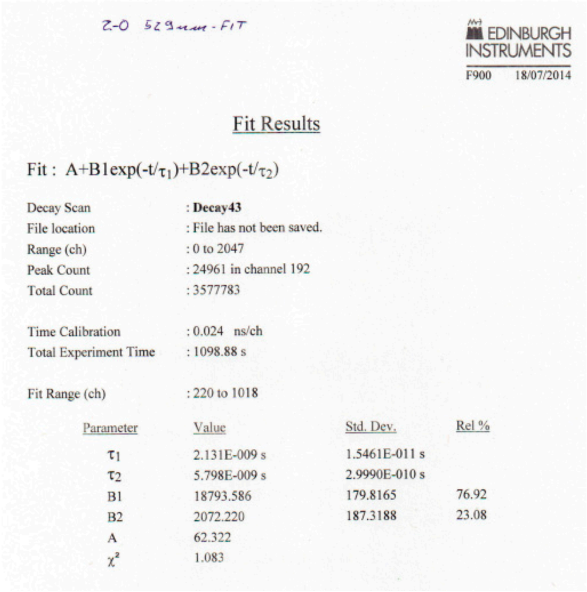

2-0 529nm-FIT

EDINBURGH INSTRUMENTS

F900 18/07/2014

Fit Results

Fit : $A+B1\exp(-t/\tau_1)+B2\exp(-t/\tau_2)$

Decay Scan	: Decay43
File location	: File has not been saved.
Range (ch)	: 0 to 2047
Peak Count	: 24961 in channel 192
Total Count	: 3577783
Time Calibration	: 0.024 ns/ch
Total Experiment Time	: 1098.88 s
Fit Range (ch)	: 220 to 1018

Parameter	Value	Std. Dev.	Rel %
τ_1	2.131E-009 s	1.5461E-011 s	
τ_2	5.798E-009 s	2.9990E-010 s	
B1	18793.586	179.8165	76.92
B2	2072.220	187.3188	23.08
A	62.322		
χ^2	1.083		

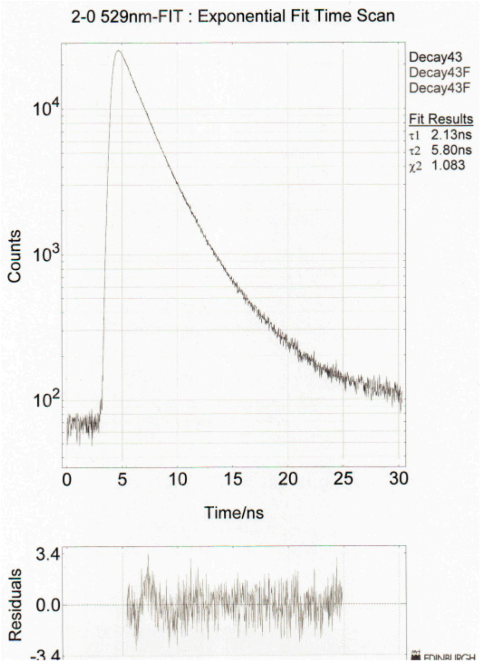

Figure S23. Relevant data from time-resolved fluorescence spectroscopy (left) and time decay curve and respective residuals from DEA3HF:R6G molar ratio=0.1 in ethanol. (Excitation wavelength: 405 nm; monitored emission wavelength: 529 nm).

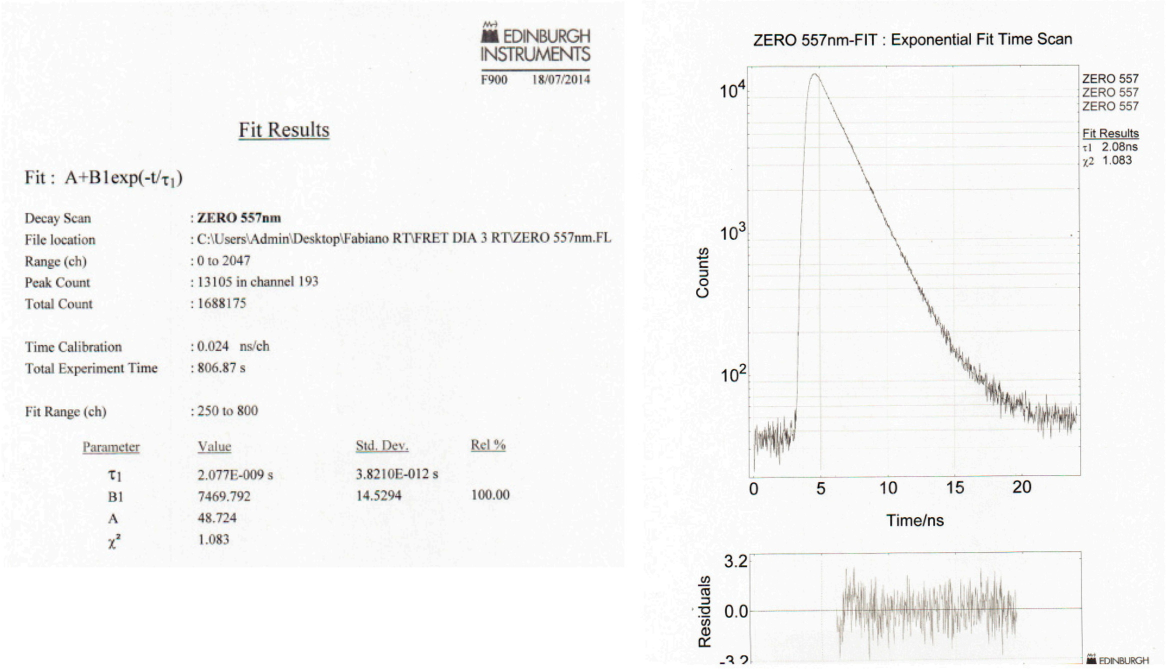
EDINBURGH INSTRUMENTS
F900 18/07/2014

Fit Results

Fit : A+B1exp(-t/τ_1)

Decay Scan : ZERO 557nm
File location : C:\Users\Admin\Desktop\Fabiano RT\FRET DIA 3 RT\ZERO 557nm.FL
Range (ch) : 0 to 2047
Peak Count : 13105 in channel 193
Total Count : 1688175

Time Calibration : 0.024 ns/ch
Total Experiment Time : 806.87 s

Fit Range (ch) : 250 to 800

Parameter	Value	Std. Dev.	Rel %
τ_1	2.077E-009 s	3.8210E-012 s	
B1	7469.792	14.5294	100.00
A	48.724		
χ^2	1.083		

Figure S24. Relevant data from time-resolved fluorescence spectroscopy (left) and time decay curve and respective residuals from DEA3HF in ethanol. (Excitation wavelength: 405 nm; monitored emission wavelength: 557 nm).

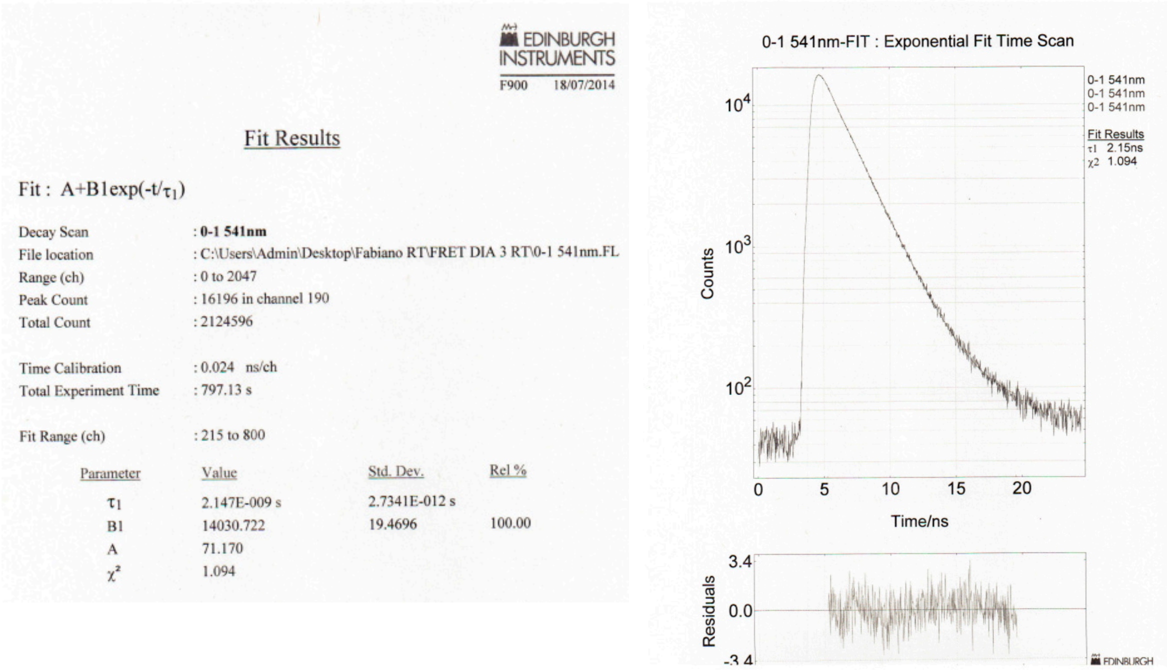
EDINBURGH INSTRUMENTS
F900 18/07/2014

Fit Results

Fit : A+B1exp(-t/τ_1)

Decay Scan : 0-1 541nm
File location : C:\Users\Admin\Desktop\Fabiano RT\FRET DIA 3 RT\0-1 541nm.FL
Range (ch) : 0 to 2047
Peak Count : 16196 in channel 190
Total Count : 2124596

Time Calibration : 0.024 ns/ch
Total Experiment Time : 797.13 s

Fit Range (ch) : 215 to 800

Parameter	Value	Std. Dev.	Rel %
τ_1	2.147E-009 s	2.7341E-012 s	
B1	14030.722	19.4696	100.00
A	71.170		
χ^2	1.094		

Figure S25. Relevant data from time-resolved fluorescence spectroscopy (left) and time decay curve and respective residuals from DEA3HF:R6G molar ratio=2.0 in ethanol. (Excitation wavelength: 405 nm; monitored emission wavelength: 541 nm).

EDINBURGH INSTRUMENTS

F900 18/07/2014

Fit Results

Fit : $A+B1exp(-t/\tau_1)+B2exp(-t/\tau_2)$

Decay Scan	: **0-5 553nm**
File location	: C:\Users\Admin\Desktop\Fabiano RT\FRET DIA 3 RT\0-5 553nm.FL
Range (ch)	: 0 to 2047
Peak Count	: 9543 in channel 198
Total Count	: 1463778
Time Calibration	: 0.024 ns/ch
Total Experiment Time	: 924.15 s
Fit Range (ch)	: 210 to 1030

Parameter	Value	Std. Dev.	Rel %
τ_1	2.196E-009 s	4.2299E-011 s	
τ_2	4.529E-009 s	2.6897E-010 s	
B1	7371.433	290.6757	66.52
B2	1798.740	298.4575	33.48
A	56.909		
χ^2	0.965		

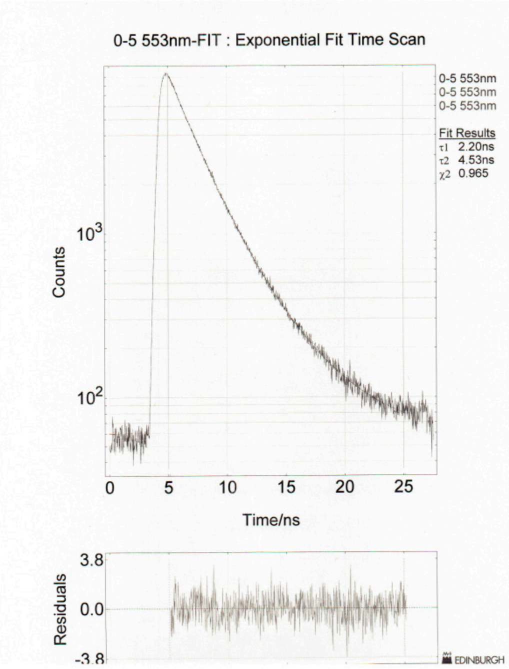

Figure S26. Relevant data from time-resolved fluorescence spectroscopy (left) and time decay curve and respective residuals from DEA3HF:R6G molar ratio=1.5 in ethanol. (Excitation wavelength: 405 nm; monitored emission wavelength: 553 nm).

EDINBURGH INSTRUMENTS

F900 18/07/2014

Fit Results

Fit : $A+B1exp(-t/\tau_1)+B2exp(-t/\tau_2)$

Decay Scan	: **1-0 556nm**
File location	: C:\Users\Admin\Desktop\Fabiano RT\FRET DIA 3 RT\1-0 556nm.FL
Range (ch)	: 0 to 2047
Peak Count	: 7771 in channel 201
Total Count	: 1376296
Time Calibration	: 0.024 ns/ch
Total Experiment Time	: 953.43 s
Fit Range (ch)	: 210 to 1148

Parameter	Value	Std. Dev.	Rel %
τ_1	2.237E-009 s	6.6851E-011 s	
τ_2	4.390E-009 s	1.3723E-010 s	
B1	4607.637	297.7338	43.26
B2	3079.303	305.9093	56.74
A	60.728		
χ^2	1.082		

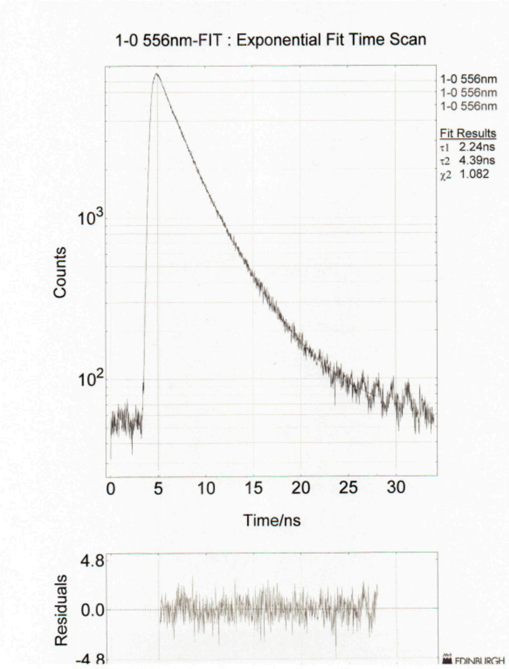

Figure S27. Relevant data from time-resolved fluorescence spectroscopy (left) and time decay curve and respective residuals from DEA3HF:R6G molar ratio=1.0 in ethanol. (Excitation wavelength: 405 nm; monitored emission wavelength: 556 nm).

EDINBURGH INSTRUMENTS
F900 18/07/2014

Fit Results

Fit : $A+B1\exp(-t/\tau_1)+B2\exp(-t/\tau_2)$

Decay Scan	: **1-5 557nm**
File location	: C:\Users\Admin\Desktop\Fabiano RT\FRET DIA 3 RT\1-5 557nm.FL
Range (ch)	: 0 to 2047
Peak Count	: 12580 in channel 194
Total Count	: 2312981
Time Calibration	: 0.024 ns/ch
Total Experiment Time	: 682.00 s
Fit Range (ch)	: 200 to 1305

Parameter	Value	Std. Dev.	Rel %
τ_1	2.341E-009 s	5.3139E-011 s	
τ_2	4.564E-009 s	6.8397E-011 s	
B1	6537.203	305.0057	35.08
B2	6206.558	315.5979	64.92
A	58.642		
χ^2	1.077		

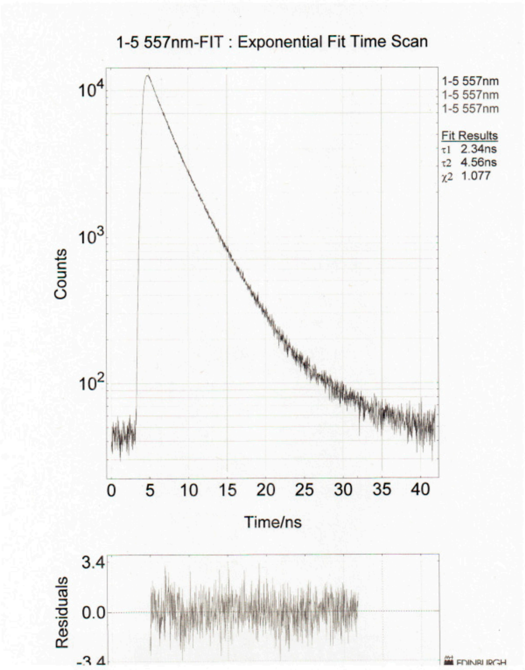

Figure S28. Relevant data from time-resolved fluorescence spectroscopy (left) and time decay curve and respective residuals from DEA3HF:R6G molar ratio=0.5 in ethanol. (Excitation wavelength: 405 nm; monitored emission wavelength: 557 nm).

EDINBURGH INSTRUMENTS
F900 18/07/2014

Fit Results

Fit : $A+B1\exp(-t/\tau_1)+B2\exp(-t/\tau_2)$

Decay Scan	: **2-0 559nm**
File location	: C:\Users\Admin\Desktop\Fabiano RT\FRET DIA 3 RT\2-0 559nm.FL
Range (ch)	: 0 to 2047
Peak Count	: 11040 in channel 201
Total Count	: 2305916
Time Calibration	: 0.024 ns/ch
Total Experiment Time	: 1224.89 s
Fit Range (ch)	: 220 to 1080

Parameter	Value	Std. Dev.	Rel %
τ_1	2.574E-009 s	1.1581E-010 s	
τ_2	4.988E-009 s	1.7164E-010 s	
B1	4871.569	545.7723	31.60
B2	5442.681	551.9162	68.40
A	83.491		
χ^2	1.032		

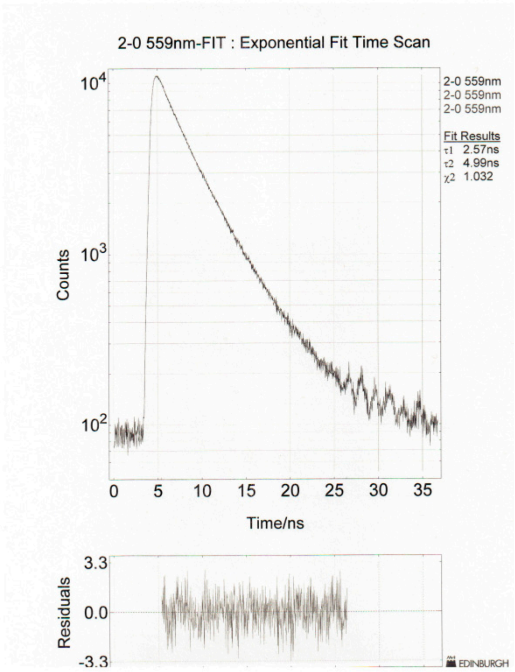

Figure S29. Relevant data from time-resolved fluorescence spectroscopy (left) and time decay curve and respective residuals from DEA3HF:R6G molar ratio=0.1 in ethanol. (Excitation wavelength: 405 nm; monitored emission wavelength: 559 nm).

Table S7. Working concentration for Stern-Volmer plot.

System	Molar Ratio	Concentration (10^{-6} mol/L)	
		Donor	Acceptor
		DEA3HF	R6G
DEA3HF@$(OA)_2$	0	15.6	0
	2.0		1.58
	1.5		7.92
	1.0		15.8
	0.5		23.8
	0.1		31.7
DEA3HF in ethanol	0	5.20	0
	2.0		0.53
	1.5		2.63
	1.0		5.27
	0.5		7.90
	0.1		10.5

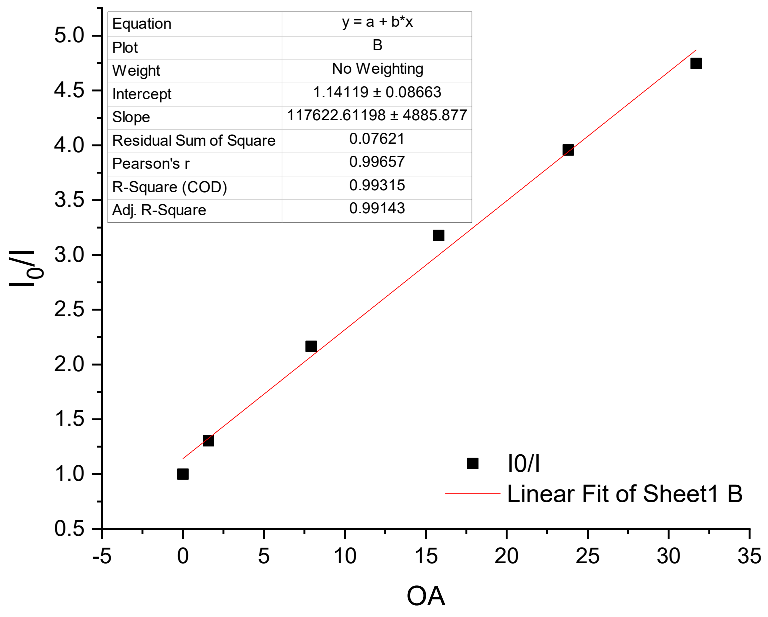

Figure S30. Stern-Volmer plot of DEA3HF@$(OA)_2$ in sodium tetraborate buffer in the presence of different amounts of R6G. The parameters from the linear fit are also presented for calculation purposes.

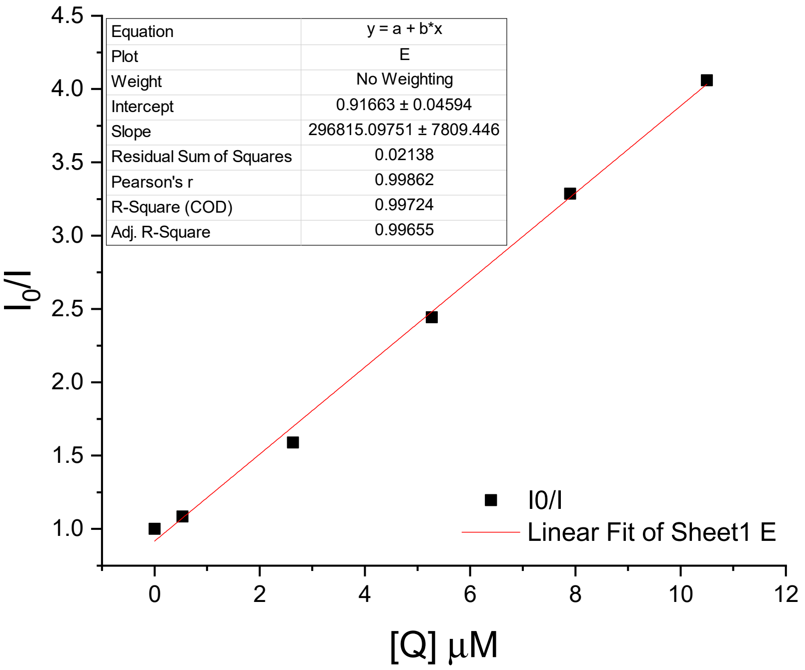

Figure S31. Stern-Volmer plot of DEA3HF in ethanol in the presence of different amounts of R6G. The parameters from the linear fit are also presented for calculation purposes.
